# Unspoken cycles: A qualitative study exploring the lived experiences of women’s menstruation in the urban informal settlements of Bangladesh

**DOI:** 10.1371/journal.pone.0354260

**Published:** 2026-07-24

**Authors:** Marufa Alam, Md. Al-Mamun

**Affiliations:** 1 Department of Public and Community Health, Faculty of Medicine and Health Sciences, Frontier University Garowe, Puntland, Somalia; 2 BRAC Institute of Governance and Development (BIGD), BRAC University, Dhaka, Bangladesh; Cranfield University, UNITED KINGDOM OF GREAT BRITAIN AND NORTHERN IRELAND

## Abstract

**Background:**

Menstrual health and hygiene (MHH) is a critical component of public health and gender equity. However, limited research has explored the everyday experiences of women and adolescent girls managing menstruation within the constrained environment of urban informal settlements. This study explores the lived experiences of menstruation among women and girls in the Khulna Railway Slum in Bangladesh.

**Methods:**

Guided by a Feminist Political Ecology framework, this qualitative study employed a combined inductive and deductive thematic approach. Data were collected between September and October 2025 through in-depth interviews with 18 women and adolescent girls aged 15–45 years living in urban informal settlements in Khulna. Five key informant interviews were also conducted with relevant stakeholders. Data were analyzed thematically using both pre-defined conceptual domains and emergent codes.

**Findings:**

Findings revealed that menstrual experiences are shaped by three interrelated conceptual domains: structural-environmental constraints, socio-cultural stigma, and gendered inequities in access to resources. Inadequate WASH facilities, poor waste management, and lack of affordable menstrual products hinder safe and dignified menstrual practices. Stigma and taboos reinforced shame, exclusion, and silence, while gendered inequalities limited autonomy and resource access, especially among adolescents.

**Conclusion:**

The study highlights menstruation as a socially and structurally shaped experience influenced by infrastructural constraints, cultural norms, and gendered inequalities. It contributes to scholarship on menstrual health by emphasizing the need to address menstrual inequities as part of broader efforts to improve gender equity and WASH services in urban informal settlement.

## Introduction

Menstrual health and hygiene (MHH) are recognized as a fundamental aspect of public health, gender equity, and social well-being; yet, MHH remain a largely neglected area, particularly in low-income urban settlements [1–3]. Menstruation is not merely a biological event; it is deeply embedded in socio-cultural norms, beliefs, and practices that shape the everyday experiences of women and adolescent girls [4]. Globally, inadequate menstrual hygiene and health are linked to reproductive tract infections (RTIs), urinary tract infections (UTIs), anemia, and other complications, as well as psychological distress, anxiety, and social exclusion [5–7]. Ensuring equitable access to safe menstrual products, private sanitation, and a reliable water supply has thus become central to achieving Sustainable Development Goals (SDGs) 3 (health), 4 (education), 5 (gender equality), and 6 (clean water and sanitation) [8–10].

In South Asia, MHH challenges are intensified by poverty, entrenched gender norms, and inadequate WASH (water, sanitation, and hygiene) infrastructure [10,11]. Evidence from India, Nepal, and Pakistan reveals that women and adolescent girls in low-resource urban and rural areas face obstacles like restricted mobility during menstruation, limited access to affordable products, and unhygienic sanitation facilities, leading to exclusion, absenteeism, and health risks [2,12,13]. Cultural taboos and social stigma further silence menstruation-related discussions and restrict awareness about hygiene and reproductive health [1,5,11].

Bangladesh’s urban informal settlements, or slums, exemplify these challenges vividly. These densely populated areas, such as slums, are characterized by poor infrastructure, limited sanitation, and restricted access to healthcare [3,14]. Khulna, a major city in the southwest, is home to many informal settlements where structural, economic, and environmental constraints intensify menstrual health vulnerabilities [15]. Shortages of clean water, absence of private toilets, and unaffordability of sanitary products are compounded by cultural stigmas and taboos [16,17].

Prior studies indicate that adolescent girls and women in urban slums often struggle to manage menstruation safely. In Dhaka, for example, 59% of adolescent girls reported using old clothes due to financial constraints, reflecting economic and accessibility challenges [18]. In Rajshahi, 82% of urban girls used sanitary pads, but usage declined sharply among lower-income families [4].

Cultural beliefs further shape menstrual experiences in Bangladesh. Menstruation remains shrouded in silence, myths, and taboos, limiting open discussion [4,19,20]. In some communities, menstruating women are prohibited from entering kitchens, attending religious events, or interacting with men, restrictions rooted in deep-seated gender hierarchies [20,21]. Women and adolescent girls often experience shame, embarrassment, and fear during menstruation, which limits their involvement in home, educational, and community activities [3,6]. These practices not only cause emotional distress but also reinforce structural gender inequality by constraining women’s educational, economic, and civic engagement. Menstrual stigma is linked to absenteeism, diminished academic performance, and reduced social participation [1,22]. Adult women similarly face restricted mobility, lack of private sanitation, and increased exposure to harassment and gender-based violence during menstruation [5,23]. Cultural silence surrounding menstruation often hinders substantive discourse on challenges or health concerns, while myths and misconceptions perpetuate detrimental practices, such as using unsanitary materials or refraining from bathing during menstruation [4,20,24]. These experiences demonstrate that MHH is not merely a biological concern but also a socio-cultural phenomenon influenced by gendered norms, power dynamics, and social structures [25, 26].

Environmental and infrastructural vulnerabilities heighten these challenges in Khulna’s informal settlements. Overcrowding, inadequate drainage, and limited access to water hinder proper menstrual hygiene management [3,19]. These settlements are also prone to floods, cyclones, and tidal surges, which disrupt WASH services and exacerbate sanitation insecurity [15,27]. Women and girls often resort to unsafe practices like using makeshift cloths, open areas, or overcrowded public toilets raising the risk of RTIs and UTIs [5,17]. Financial constraints further restrict the ability to purchase sanitary products, compounding the problem [4].

Recognizing MHH as a multifaceted issue, interventions in Bangladesh and other LMICs have sought a blend of education, product accessibility, and sanitation enhancement. Menstrual education programs in schools in Dhaka and Chittagong have helped teenage girls learn more about their menstrual health and maintain personal hygiene [1,22]. Community-based programs that distribute affordable sanitary pads have also made it easier for people in urban slums to access them [17]. These initiatives often fail to adequately target adult women, out-of-school adolescents, and infrastructural deficiencies, such as the absence of private toilets, inadequate water supply, and insufficient waste disposal facilities [5,19]. Additionally, socio-cultural stigma and gender norms persist in obstructing the adoption of safe practices, underscoring the necessity for integrated methods that tackle both material and social determinants of menstrual health and hygiene (MHH) [17–20].

Studies from South Asia highlight that unsafe MHH practices are associated not only with physical health risks but also with psychosocial stress, school absenteeism, and reduced social participation [1,6,7]. Women’s limited access to water, sanitation, and affordable menstrual products interacts with social taboos to reinforce cycles of exclusion and marginalization [3,4,28]. In addition, environmental vulnerabilities in coastal regions, such as flooding and water salinity, amplify barriers to hygienic menstrual management [15,19], further underscoring the need for context-specific research in Khulna’s informal settlements.

Prior research on MHH in Bangladesh has predominantly concentrated on school-based interventions or rural populations [4,17], resulting in the under-representation of adult women, out-of-school girls, and residents of urban informal settlements. A limited number of studies have comprehensively investigated the interplay of socio-economic, infrastructural, and cultural elements in influencing menstruation patterns, coping strategies, and access to hygiene supplies in informal urban environments [3,13,20]. Moreover, the lived experiences, beliefs, and coping mechanisms of women and teenage girls managing menstrual health and hygiene in high-density, low-resource metropolitan areas are predominantly unrecorded. To create culturally sensitive, context-specific, and inclusive interventions, we need to address these gaps. These interventions aim to enhance menstrual dignity, health, and gender equality.

By integrating previous findings on MHH in urban slums, this study situates menstrual experiences within broader structural and socio-cultural contexts. The present study addresses these gaps by focusing on the informal settlements of Khulna Railway Slum, exploring the complex socio-cultural, economic, and infrastructural dimensions of MHH. The research aims to understand the barriers women and adolescent girls face in accessing safe menstrual hygiene facilities and products, examine the socio-cultural stigmas surrounding menstruation, and identify coping strategies employed in response to these challenges. In this paper we discuss women’s day-to-day experiences with managing menstruation in environments where services for water, sanitation, and waste management and disposal are limited and social and gender inequities are prevalent. By centering the voices of women and girls living in these settlements, the study provides a nuanced understanding of the multidimensional challenges affecting MHH and informs evidence-based, context-specific interventions.

### Theoretical framework

Using the Feminist Political Ecology (FPE) paradigm as a guide, this research explores the complex ways in which the menstrual health of urban underprivileged women is influenced by the interplay of gender, power, and environmental factors. Environmental and health disparities are not viewed solely as technical difficulties, but rather as socially and politically constructed. This view is held by FPE, which emerged from the confluence of feminist theory and political ecology [25,29]. This highlights the fact that water, sanitation, and waste management systems are influenced by gender, class, and culture [30,31].

Using the FPE paradigm, we can observe how gendered vulnerabilities are exacerbated in informal settlements by structural and environmental restrictions. Periods are especially difficult for women and girls in communities where water is scarce, sanitation is poor, or there are no facilities to dispose of human waste. According to [32] and [33], these shortcomings are not coincidental but rather reflect larger systemic injustices associated with poverty, urban marginalization, and state neglect. As a result, the socio-ecological contexts in which women live are crucial to understanding their reproductive and menstrual health, as these contexts frequently compel women to deal with menstruation in hazardous, unsanitary, and degrading settings.

Patriarchal discourse governs female bodies and inhibits women’s participation in both household and public arenas [11,34]. The FPE framework also highlights the socio-cultural shame surrounding menstruation. As a result of menstruation being stigmatized or ignored, many women experience embarrassment, limited movement, and social marginalization [31,35]. According to FPE, these taboos are more than just cultural relics; these systems perpetuate gendered power dynamics, keeping women subordinate and unheard in decision-making [36]. Patriarchal standards of modesty and silence keep women’s bodily needs second, and the stigma associated with menstruation only serves to perpetuate this imbalance.

Last but not least, the framework highlights the ways in which economic insecurity, social exclusion, and gender hierarchies’ impact menstrual health management, as well as gendered disparities in access to resources. Household priorities generally prioritize male or collective needs above women’s personal hygiene, and adolescent girls and women in slum settings sometimes lack the financial liberty to buy period products [1,37]. Gender norms and poverty both contribute to the exclusionary cycle. Because of their dependence and lack of information, teenage girls face even greater vulnerabilities. According to FPE theory, these disparities are the result of systemic oppression that views menstruation as a social injustice as well as a health problem [26,29].

**Fig 1:**
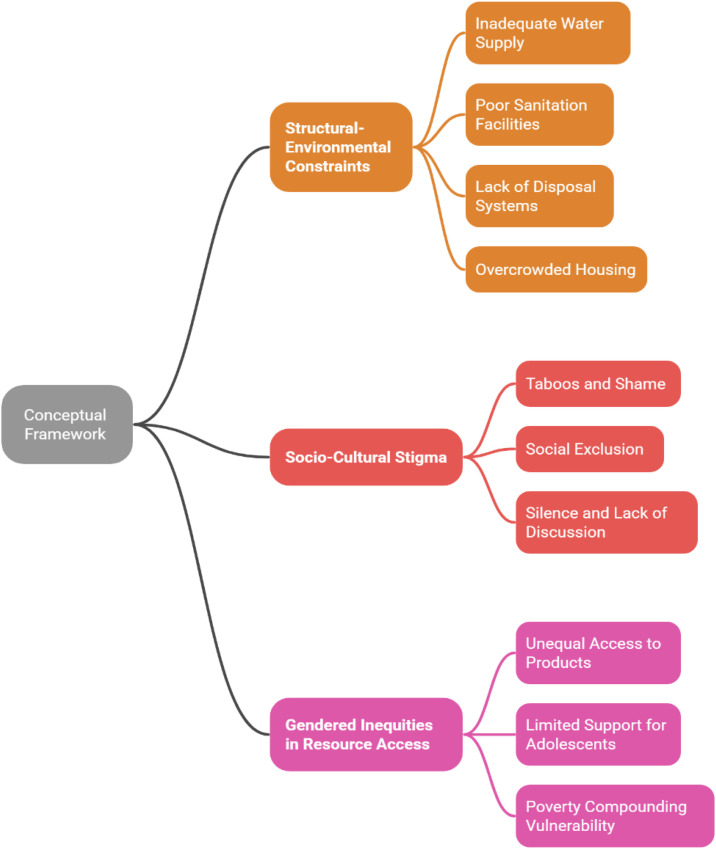
Conceptual framework based on Feminist Political Ecology (FPE).

Accordingly, this study adopts Feminist Political Ecology as its guiding theoretical framework to explore the lived experiences of menstrual health among women and adolescent girls residing in informal settlements. The three conceptual domains: (1) Structural-Environmental Constraints, (2) Socio-Cultural Stigma, and (3) Gendered Inequities in Resource Access—collectively provide an analytical structure for understanding how environmental scarcity, cultural taboos, and economic deprivation interact to shape menstrual practices and well-being (See Fig 1). By applying FPE, this study not only reveals the unequal power relations embedded in menstrual health but also challenges the structural and cultural systems that sustain gendered marginalization in urban poverty contexts.

## Materials and methods

### Research design

Using the Consolidated Criteria for Reporting Qualitative Research (COREQ) as a guide [38], this qualitative study examines the experiences of menstruation among women and girls residing in the informal settlements of Bangladesh’s Khulna Railway Slums. The study is based on the interpretivist research paradigm. We followed a deductive approach as our research methodology. Using an existing theory or hypothesis as a starting point, qualitative researchers employ a deductive strategy to gather and analyze data [39]. To provide a solid groundwork for our research, we employed feminist political ecology to guide our data collection and analysis [25]. Two well-established qualitative research methodologies, in-depth interviews (IDIs) and key informant interviews (KIIs), were employed for this work. These methods offer valuable insights that have been previously mentioned [40,41]. We employed a qualitative approach to conduct the study because it facilitates the comprehension of meaningful and logical explanations of human actions, thinking, and behavior, based on subjective opinions, experiences, and judgments [42,43]. Additionally, it enables the recording of real-life stories that are shaped by complex social, political, and cultural factors [44].

### Study area

This study was conducted in Khulna, the third-largest city in Bangladesh, with a population of approximately 2.61 million in the Khulna district [16], of which 20% reside in slums [14]. There are approximately 520 informal settlements in Khulna at present [14,15]. The Railway Slum is one of the largest, situated on land owned by the Bangladesh Railway Authority (BRA). The former ward councilor and the city mayor helped set up this slum in 2002. The authority helped some homeless migrants from other coastal locations of Khulna who were living on the water. The Railway Slum is one of Khulna City’s most densely populated slums [16]. It is on the bank of the Bhairav River and is part of Khulna City Corporation’s administrative ward no. 21. The slum is bordered on the east by Upper Jessore Road, on the west by the Bhairav River, on the north by Khalishpur, and on the south by the railway station and the BIWTA ghat.

Montu Colony, Greenland Slum, and Sweeper Colony are the three segments that make up the total settlement. The Greenland slum (a large portion of the Khulna Railway Slum) was chosen because it has a population of 5,806 and a male-to-female ratio of 30:70 [14]. It is a good example of limited access to better water and sanitation services, as well as poor healthcare and rehabilitation services. Second, this slum has a higher percentage of women and girls than other slums in Khulna. Additionally, social and environmental obstacles exacerbate existing menstrual hygiene practices, increasing vulnerability and hindering all sorts of activities of women and girls.

### Ethics approval and consent to participate

This study adhered to established ethical principles for research involving human participants. Ethical approval was obtained from the Institutional Review Board (IRB) of the Department of Sociology at Gopalganj Science and Technology University, Bangladesh (Approval No. 473478-FY 2024–2025; Approval Date: 10 August 2025). The study was conducted in accordance with the principles outlined in the Declaration of Helsinki and relevant national ethical guidelines (World Medical Association, 2013).

Given the sensitive nature of menstruation and the inclusion of adolescent participants, particular attention was paid to informed consent procedures and participant protection. Adult participants (18 years and above) provided written informed consent before participation. For adolescent participants (aged below 18 years), written consent was obtained from a parent or legal guardian, alongside assent from the adolescent participant prior to the interview. All participants received clear oral and written explanations regarding the study objectives, procedures, potential risks and benefits, confidentiality measures, and their right to decline participation or withdraw from the study at any stage without any negative consequences.

To ensure privacy and confidentiality, interviews were conducted in settings chosen by participants where conversations could be held privately and comfortably. Participants’ names and other identifying information were removed from transcripts and replaced with pseudonyms. Unique identification codes were assigned to all participants to maintain anonymity throughout data management and analysis. Audio recordings, transcripts, and related research materials were stored in password-protected digital storage accessible only to the research team. These measures were implemented to protect participants’ privacy, dignity, and data security throughout the research process.

### Study population and sampling

A total of 18 participants, aged 15–45 years, were recruited using a combination of purposive and limited convenience sampling. Purposive sampling served as the primary strategy to identify information-rich participants who met the study’s inclusion criteria and represented diverse menstrual experiences across different age groups, marital status, and livelihood conditions. This approach enabled the inclusion of both adolescent girls and adult women whose experiences reflected the social and environmental diversity of the informal settlement. Limited convenience sampling was employed only during the initial stage of recruitment to facilitate access to eligible participants within the densely populated settlement, where household mobility, work schedules, and participant availability posed practical challenges. After initial contact, subsequent recruitment continued purposively to ensure diversity and relevance to the study objectives. The combination of these approaches balanced methodological rigor with the practical realities of conducting qualitative research in a resource-constrained urban informal settlement.

Participants were identified with the assistance of local community leaders and female volunteers familiar with the slum areas. Inclusion criteria included (i) women and adolescent girls of reproductive age (15–45 years), (ii) residing in the selected informal settlements for at least three consecutive years, and (iii) willingness to discuss menstrual experiences openly. To ensure ethical and analytical clarity, participants were stratified during recruitment into two groups: adolescent girls (15–19 years) and adult women (20–45 years), allowing age-sensitive interpretation of menstrual experiences.

Exclusion criteria were based on gender and age, as the study specifically aimed to capture the perspectives of women and girls. The criteria for KIIs participants were (i) community health workers or NGO staff engaged in reproductive or menstrual health programs in Khulna; (ii) local schoolteachers or social workers directly involved with adolescent girls’ welfare; and (iii) community leaders or female ward representatives familiar with sanitation, gender, or health issues in the slum areas.

### Data collection and analysis

Data collection was conducted by the authors, with the assistance of four female trained research assistants, among participants in the informal railway slum settlements of Khulna, Bangladesh. Participant recruitment and data collection were carried out between 01/09/2025 and 31/10/2025. A total of 18 in-depth interviews (IDIs) were conducted with women and teenage girls, and five Key Informant Interviews (KIIs) were conducted with community health workers, NGO officers, and schoolteachers who are engaged in menstrual or reproductive health matters.

We employed semi-structured interview schedules designed to meet the study’s objectives and aligned with the Feminist Political Ecology framework, as well as informed by established literature on menstrual health and gendered vulnerabilities in resource-limited settings [1–5,29,33]. The research complied with ethical standards as specified by the Declaration of Helsinki [45]. Data collection took place at participants’ homes, workplaces, or nearby community centers, depending on their convenience and comfort. Prior to recruitment, potential participants were informed about the study objectives, voluntary nature of participation, confidentiality measures, and their right to refuse participation without consequences.

Data collection and analysis were conducted concurrently to allow continuous assessment of data saturation. Following each interview, preliminary coding was undertaken and compared with previously collected data to identify whether new codes, concepts, or variations in participants’ experiences were emerging. By the fifteenth in-depth interview, the coding process yielded no substantially new concepts, and participants’ accounts consistently reinforced the existing thematic framework. Three additional interviews were conducted to confirm that no new conceptual insights or dimensions emerged across different participant characteristics. Similarly, after the fifth key informant interview, no additional information was identified that expanded or refined the developing themes. Based on this iterative assessment, the research team concluded that thematic saturation had been achieved, and data collection was subsequently discontinued. In qualitative research, the adequacy of the sample is determined by the richness and completeness of the data rather than by statistical representativeness [46]. Therefore, the findings are intended to support analytical rather than statistical generalization, emphasizing contextual depth, conceptual understanding, and the transferability of insights to similar settings.

All interviews were conducted in Bengali to ensure linguistic accessibility and richness of participant expression. To enhance rigor, translated meanings were preserved through back-checking of selected transcripts and peer review among the research team. Additionally, the researchers monitored the individuals’ activities during the data collection process. The interviews were audio-recorded with the participants’ consent. In addition to audio recordings, field notes were documented to capture non-verbal cues, contextual observations, and reflexive notes. Each interview session lasted, on average, 25–30 minutes. Upon concluding data collection, the authors transcribed all audio recordings into English. To ensure translation accuracy, a two-step verification process was applied: (i) initial translation by the researchers and (ii) cross-checking by another bilingual member of the research team to maintain semantic consistency.

The authors meticulously verified the transcriptions for consistency prior to conducting thematic analysis [42–43]. The data were analyzed using thematic analysis following Braun and Clarke’s six-phase framework, ensuring systematic identification, coding, and refinement of themes [43,47,48]. The analysis followed a combined inductive and deductive approach. Deductively, initial coding was guided by three pre-established conceptual domains derived from the Feminist Political Ecology framework: structural-environmental restrictions, socio-cultural stigma, and gendered disparities in resource access. Inductively, new codes emerging from the data were incorporated to capture unanticipated themes. The data were coded using NVivo qualitative analysis software (v.12) [49]. The coding process was conducted independently by MA and MA-M. Following independent coding, both coders compared codebooks, discussed discrepancies, and resolved differences through consensus meetings to ensure analytical consistency and rigor. Themes were developed through iterative processes of coding, categorization, and refinement, focusing on identifying patterned meanings rather than descriptive summaries.

To enhance methodological rigor, the study incorporated COREQ-aligned trustworthiness strategies, including credibility (through prolonged engagement and triangulation of IDIs and KIIs) [50], dependability (through audit trail and codebook documentation), confirmability (through reflexive team discussions), and transferability (through thick contextual description of informal settlement settings).All authors jointly examined and approved the final coding to ensure a thorough understanding of the findings. To preserve anonymity, individuals were assigned codes such as IDI1 and IDI2 (see Table 1) and KII1 and KII2 (see Table 2).

**Table 1:** Profile of the participants (IDIs).

Participant ID	Age Group (years)	Level of Education	Marital Status	Occupation	Length of Residence in the Community
IDI-1	15–19	Secondary incomplete	Unmarried	Student	5–9 years
IDI-2	20–24	Primary complete	Married	Garment worker	10–14 years
IDI-3	25–29	No formal education	Married	Housewife	10–14 years
IDI-4	15–19	Secondary student	Unmarried	Student	5–9 years
IDI-5	30–34	Primary incomplete	Married	Domestic worker	10–14 years
IDI-6	20–24	Secondary complete	Married	Small vendor	5–9 years
IDI-7	35–39	No formal education	Widowed	Day laborer	≥15 years
IDI-8	40–45	Primary complete	Married	Housewife	≥15 years
IDI-9	15–19	Secondary student	Unmarried	Student	5–9 years
IDI-10	25–29	Secondary complete	Married	Tailor	5–9 years
IDI-11	30–34	No formal education	Married	Housewife	10–14 years
IDI-12	20–24	Secondary complete	Married	Beauty parlor worker	5–9 years
IDI-13	35–39	Primary complete	Married	Vegetable seller	10–14 years
IDI-14	15–19	Secondary student	Unmarried	Student	5–9 years
IDI-15	40–45	No formal education	Married	Housewife	≥15 years
IDI-16	25–29	Secondary incomplete	Married	Garment worker	5–9 years
IDI-17	30–34	Primary complete	Divorced	Domestic worker	10–14 years
IDI-18	25–29	Secondary complete	Married	Street vendor	5–9 years

**Note:** To protect participant confidentiality, ages and duration of residence are presented in categories rather than as exact values, in accordance with the journal’s data-sharing requirements.

**Table 2:** Profile of the key informants (KIIs).

KII ID	Position/Role	Organization/Institution	Years of Experience	Area of Expertise	Relevance to Study
*KII-1*	Community Health Worker	Local NGO (Khulna)	6 years	Reproductive & menstrual health	Works directly with adolescent girls
*KII-2*	School Teacher	Government School	8 years	Adolescent health education	Provides insight into menstrual taboos at school
*KII-3*	NGO Program Officer	Urban Health Project	10 years	WASH & gender issues	Manages MHM awareness programs
*KII-4*	Community Leader	Railway Slum Committee	7 years	Women’s welfare	Offers local perspective on sanitation challenges
*KII-5*	Social Worker	Local Community Based Organization (CBO)	5 years	Gender and community outreach	Works with low-income women and girls

### Reflexivity

This study acknowledges that the researchers’ disciplinary backgrounds, prior engagement with gender and health research, and use of Feminist Political Ecology (FPE) as the guiding framework could influence data collection and interpretation. Therefore, reflexivity was integrated throughout the research process to critically examine how researchers’ perspectives shaped analytical decisions.

During fieldwork, the lead researcher maintained reflexive field notes and journals after each interview to document methodological observations, personal reflections, emerging interpretations, and potential assumptions. Rather than serving solely as a record of field experiences, these notes were revisited during analysis to examine whether preliminary interpretations reflected participants’ accounts or were influenced by researchers’ prior knowledge and theoretical expectations.

Regular analytical discussions were held among the research team throughout coding and theme development. During these meetings, preliminary interpretations were compared with the original interview transcripts, and researchers critically questioned whether emerging themes were sufficiently supported by participants’ narratives. Where interpretations appeared to reflect the assumptions of the guiding theoretical framework more than the participants’ own accounts, the transcripts were revisited, codes were refined, and supporting quotations were re-examined before themes were finalized. This iterative process also informed decisions about retaining, merging, or redefining codes to ensure that themes represented recurring patterns evident across participants’ narratives rather than researchers’ expectations.

Throughout the analytical process, reflexivity functioned as an ongoing practice of questioning interpretations, revisiting the data, and ensuring that participant’s lived experiences remained central to the development of the findings. This approach enhanced the credibility, dependability, and transparency of the analysis.

## Findings

### Characteristics of the participants

The study participants (see Table 1) consisted of women and adolescent girls aged 15–45 years. The participants’ ages ranged from 15 to 45 years, with the predominant segment (39%) falling within the 20- to 30-year range, indicating a focus on the reproductive age group most affected by menstrual health issues. Regarding education, the majority of participants were either non-literate or had minimal schooling (about 39%), while 33% had attained secondary education and 28% had completed primary education. The prevalent marital status among participants was married (67%), followed by unmarried (22%), widowed (6%), and divorced (6%). The majority of participants were employed in low-income informal occupations, like domestic assistance, vending, tailoring, or garment production. Approximately 28% were homemakers, 17% were domestic or textile workers, 11% were small-scale sellers or day laborers, and 22% were students. These professional profiles illustrate their economic vulnerability and limited access to menstrual hygiene options. Concerning settlement patterns, the majority (72%) had been in the slum districts for over eight years, while the remainder had lived there for five to seven years. Their prolonged residency subjected women to ongoing difficulties related to insufficient sanitation, overcrowding, and ineffective waste management, influencing their menstrual health practices.

### Emerging themes from informal settlements experiences with menstruation

The themes and sub-themes that emerged from this study are compiled in Table 3. Three major themes emerged in this study, which guided the result section: structural-environmental constraints, socio-cultural stigma, and gendered inequities in access to menstrual resources.

**Table 3:** Summarized themes, sub-themes, and codes.

Themes	Sub-Themes	Illustrative Codes
Structural–Environmental Constraints	Inadequate water supply	• Limited access to safe water sources; • dependence on shared or distant taps; • water scarcity during menstruation.
	Poor sanitation facilities	• Lack of private toilets; • shared latrines without doors or locks; • unhygienic conditions increasing infection risk.
	Lack of disposal/waste management systems	• Absence of bins; • improper disposal in drains or open spaces; • shame around discarding used materials.
	Overcrowded and unsafe housing	• Congested rooms; • lack of privacy for changing or washing; • insecurity during night-time toilet use.
Socio–Cultural Stigma	Taboos and shame around menstruation	• Menstruation viewed as impure; • feelings of embarrassment; • restricted social interactions.
	Social exclusion and restricted mobility	• Prohibition from cooking, praying, or attending gatherings; • isolation during menstrual days.
	Silence and lack of discussion in households/community	• Mothers’ reluctance to discuss menstruation; • absence of open dialogue in family or community spaces.
Gendered Inequities in Resource Access	Unequal access to menstrual products	• Financial inability to purchase sanitary pads; • reliance on old cloths or rags; • inconsistent product availability.
	Limited support for adolescents	• Inadequate menstrual education; • reliance on peers; • lack of emotional and informational support.
	Poverty compounding vulnerability	• Economic hardship restricting health-seeking behavior; • prioritization of family needs over menstrual care.

### Theme I: Structural-environmental constraints

The research indicated that women residing in informal settlements of Khulna Railway Slum encounter numerous structural and environmental obstacles that significantly impair their capacity to manage menstruation with dignity. Insufficient water and sanitation facilities, ineffective waste management systems, and congested housing circumstances collectively create a daily experience of misery, humiliation, and health hazards. These difficulties are fundamentally entrenched in systemic neglect, where gender, poverty, and urban inequality converge to influence women’s lives.

#### Inadequate water supply.

Water scarcity emerged as a prevalent and troubling concern expressed by participants. Most women reported enduring prolonged waits to obtain water from communal tube wells or public taps, occasionally returning home with insufficient water for domestic tasks and personal hygiene. These experiences demonstrate that availability to water is a crucial factor in ensuring menstrual dignity within underprivileged populations. During menstruation, this deficiency became very severe, rendering them incapable of maintaining personal hygiene or laundering menstrual linens. One woman explained,

“When water stops coming from the tap, I wait for hours. If it doesn’t come, I can’t clean myself properly. Sometimes, the cloth smells bad, and I feel uneasy all day.” *(IDI-08, Housewife)*

This quotation encapsulates both the physical challenges and the mental weight associated with menstruation hygiene. Many participants articulated their prioritization of home water requirements, such as cooking and dishwashing, over personal hygiene, highlighting how gender roles and domestic obligations exacerbate inequity. Another participant shared,

“We often manage with little water. I wash my clothes quickly so that others can use the rest. If I use too much, they scold me.” *(IDI-11, housewife)*

Such narratives reveal that water scarcity is not merely a technical issue but a gendered one. Women internalize the scarcity, rationing their hygiene to avoid family conflict or community criticism. A local NGO representative reinforced this, noting,

“For poor women in slum areas, water is a luxury. They manage their menstruation with whatever is left after other uses.” *(KII-03, NGO Program Officer)*

#### Poor sanitation facilities.

The majority of participants reported that the accessible bathrooms, which were communal among several families, were deficient in terms of privacy, safety, and hygiene. Women frequently refrained from utilizing restrooms during menstruation due to fear, embarrassment, or discomfort. Consequently, sanitary issues during menstruation pertained not only to physical accessibility but also to the overarching disregard for women’s comfort, dignity, and safety. The lack of locks, sufficient illumination, or designated areas for women rendered menstruation management a perpetual cause of distress. A 15-year-old girl explained,

“The toilet near our room has no door. Boys and men walk by all the time. I feel scared to change my clothes there.” *(IDI-04, student)*

For many adolescent girls, the lack of private sanitation facilities contributed to absenteeism from school during menstruation and reinforced feelings of embarrassment. Another participant shared a similar experience:

“At night, the toilets are far and dark. I don’t go out then. I use a bucket inside the room and throw the water away in the morning.” *(IDI-03, housewife)*

These circumstances underscore the inadequacy of sanitation facility design and placement in addressing the special needs of women. Women frequently articulated concerns over harassment, especially when utilizing public toilets at night. As one community leader observed,

“These toilets are made for everyone, but not with women in mind. Privacy and safety are missing from the very beginning.” *(KII-01, Community Health Worker)*

#### Lack of disposal and waste management systems.

Disposal of menstrual waste was one of the most overlooked facets of hygiene management in informal communities. No particular bins or trash collection systems for sanitary supplies existed, leading women to dispose of unwanted pads or cloths in drains, adjacent shrubs, or by burying them discreetly. These behaviors originated from both the lack of facilities and the pervasive cultural taboos around menstruation. One woman expressed,

“I wrap the used cloth in paper and throw it away at night. I don’t want anyone to see it; people will gossip.” *(IDI-10, tailor)*

This act of hiding menstrual materials reflects deep-rooted feelings of shame. Another respondent added,

“We have no place to throw pads. I dig a small hole behind the toilet and cover it with soil.” *(IDI-13, vegetable seller)*

Such unsafe disposal methods not only endanger health but also contaminate the environment. A key informant commented,

“Women manage their waste in secret because no one talks about it. But the drains get blocked, and children fall sick. It’s a silent crisis.” *(KII-05, Social Worker)*

#### Overcrowded and unsafe housing.

The housing conditions in informal settlements exacerbated the challenges faced by women. The majority of families resided in single-room accommodations with minimal or no privacy. Women reported a persistent sense of vulnerability and discomfort during menstruation. They frequently had difficulties in altering or drying menstruation products discreetly, apprehensive of potential embarrassment or humiliation from family members. A widowed woman described,

“We all sleep in the same small room—my sons, daughters-in-law, and grandchildren. I can’t hang my clothes openly, so I hide them behind the bed.” *(IDI-07, Day laborer)*

Such cramped spaces made menstruation management a private struggle. Another participant noted,

“Sometimes I dry the cloth under the bed, but it doesn’t dry properly. Then it smells, and I feel ashamed.” *(IDI-15, housewife)*

Women’s narratives also revealed feelings of insecurity, especially at night. Broken doors, open roofs, and poor lighting heightened their sense of vulnerability. A local leader reflected,

“These houses are too close, too unsafe. Even locking a door feels uncertain. For women, that means no peace, not even during their periods.” *(KII-03, NGO Program Officer)*

### Theme II: Socio-cultural stigma

Participants consistently described menstruation as something that should remain hidden rather than openly discussed. Their narratives showed that cultural taboos and silence surrounding menstruation influenced their daily practices, mobility, and social interactions. Many women and adolescent girls also described feelings of embarrassment, isolation, and anxiety associated with managing menstruation in the settlement.

#### Taboos and shame around menstruation.

Experiencing menstruation was emotionally fraught with shame and taboo. Menstruation was characterized by almost all participants as *lojja-r bishoy*, or something to be ashamed of and kept hidden. A code of silence was passed down through the generations as mothers warned their daughters not to talk about or show signs of menstruation. As one adolescent explained with hesitation:

“When I first started, I didn’t know what was happening. I thought I was hurt. My mother only said, ‘don’t talk about this to anyone.’ She gave me an old sari piece and told me to dry it in a dark corner.” *(IDI-4, secondary student)*

This culture of concealment reinforced internalized stigma. Several women said they felt “unclean” and “guilty” during menstruation, often isolating themselves even when not explicitly asked to. A 28-year-old woman described:

“Those days, I feel like I’m doing something wrong. If anyone sees blood on my cloth, I feel ashamed for days. I don’t even go to the shop because the men will stare………” *(IDI-3, vegetable seller)*

The taboo around menstruation also limited women’s access to menstrual products. Many participants reported feeling hesitant to purchase pads because of the fear of judgment from male shopkeepers or neighbors. One participant described her experience:

“I wanted to buy pads, but the shopkeeper laughed at me and asked why I needed them. After that, I stopped going myself and now use old cloths at home.” *(IDI-7, day laborer)*

Together, these accounts suggest that many participants felt pressure to conceal menstruation from others. Maintaining privacy and avoiding visible signs of menstruation were repeatedly described as important for avoiding embarrassment, criticism, or unwanted attention within the community.

#### Social exclusion and restricted mobility.

Participants also described several social and everyday restrictions associated with menstruation. During their periods, participants outlined a set of “unwritten rules” that limited their movement and restricted the handling of certain items. These regulations permeated all public places, including homes, schools, and places of worship. As one older woman put it:

“When I bleed, I cannot enter the kitchen or touch the water jar. My mother-in-law says if I do, the whole house becomes polluted.” *(IDI-8, housewife)*

Even outside the home, these taboos limited women’s ability to take part in society and the economy. Several women explained that they missed work during menstruation because they lacked safe toilets, water, or private spaces to manage menstrual bleeding and feared embarrassment if menstrual blood became visible. A young vendor shared:

“I sell vegetables near the station, but when I am bleeding, I can’t go. There’s no toilet, no water. If something leaks, people will laugh. I lose income but have no choice.” *(IDI-13, vegetable seller)*

Several adolescent girls reported missing school due to inadequate sanitation facilities and fear of embarrassment during menstruation. A local schoolteacher described how menstrual-related stigma and expectations within the school environment contribute to their absence:

“Girls are taught to hide everything. If blood leaks, other students mock them. Some parents also stop them from going to school during those days, saying it’s unsafe or improper.” *(KII-2, school teacher)*

#### Silence and lack of discussion in households and the community.

Participants commonly described menstruation as a private issue that was rarely discussed within households or the wider community. In most families and communities, talking about periods was taboo. Men thought it was a demeaning subject, and mothers were either too embarrassed or ignorant to teach their daughters about it. One participant reflected on the absence of communication with her own mother:

“My mother never explained anything. She just said, ‘you’re grown up now, stay away from men.’ I learned from friends, but many of them also didn’t know much.” *(IDI-9, student)*

Limited discussion within families contributed to uncertainty and misconceptions about menstruation among several participants. Some of the participants thought that touching plants would cause them to wither or that taking a bath while menstruating was harmful. These views illustrate the complex relationship between local conceptions of cleanliness and contamination and the lack of reliable information. A community NGO worker confirmed this systemic silence:

“We try to run awareness programs, but most families don’t want to attend if the topic is menstruation. They think it’s shameful. Even women whisper the word ‘period.” *(KII5, Social Worker)*

Few participants reported seeking advice from healthcare providers regarding menstrual problems. Instead, many viewed menstrual pain, irregular bleeding, or infections as a normal part of womanhood that did not require medical attention. Period issues were viewed by women as a “*normal suffering*” of womanhood, and they medical advice rarely. The lack of treatment for infections, irregular periods, and pain is a reflection of how silence may lead to health neglect.

### Theme III: Gendered inequities in resource access

Participants described unequal access to menstrual resources as an important challenge during menstruation. Their accounts highlighted how household financial constraints, gender norms, and limited community support shaped access to menstrual products and information. In order to get the menstrual products, they need, women and girls face a tangled web of family, societal, and financial barriers. Disparities like these manifested on various levels, including the distribution of menstruation products among households, the community’s failure to support teenage females, and poverty itself. These experiences suggest that limited resources were closely intertwined with household decision-making, community norms, and the broader living conditions of the informal settlement.

#### Unequal access to menstrual products.

Many participants reported that purchasing menstrual products depended on household financial priorities and decisions made by other family members, particularly male earners. A multitude of women indicated a dependence on antiquated fabrics, rags, or improvised materials, not out of preference, but due to a lack of control over household expenditures. Several participants explained that menstrual products were often considered less important than other household expenses, leading them to rely on reusable cloth instead of commercial sanitary pads. One participant stated:

“I wanted to buy pads, but my husband said it was a waste of money. I use old clothes instead. Sometimes they leak, but I’m not allowed to tell anyone. If I ask again, he scolds me. *(IDI-3, housewife)*

Participants also described feeling uncomfortable purchasing sanitary pads because they anticipated embarrassment or negative reactions from male shopkeepers and other community members. As another participant explained:

“In our slum, shopkeepers are men. If I go to buy pads, they laugh or whisper. I send my friend instead, or just use old cloth. I feel anxious every time.” (*IDI-2, garment worker*)

#### Limited support for adolescents.

Many adolescent participants reported receiving little guidance or preparation before experiencing their first menstruation. The study found that girls were not getting up-to-date knowledge regarding menstruation due to intergenerational silence and long-standing taboos.

One adolescent participant described her first experience:

“I was scared when I saw blood. My mother told me not to speak about it and gave me a torn cloth. I stayed in my room all day because I was ashamed.” *(IDI-4, secondary student)*

Periods were frequently reported as a reason for school absenteeism among adolescent girls, primarily due to lack of privacy, inadequate sanitation facilities, and feelings of shame associated with managing menstruation in school settings. A participant shared her experience:

“During my periods, I stay home because I cannot manage the clothes properly at school. The toilets are dirty, and there is no water. I miss classes every month.” *(IDI-14, secondary student)*

Key informants highlighted that this gap is systemic and affects many households:

“Many families overlook adolescent girls when distributing sanitary products or offering guidance. Society sees menstruation as private, so adolescent needs are neglected.” *(KII-4, Community Leader)*

#### Poverty compounding vulnerability.

Participants consistently described poverty as an important factor shaping how they managed menstruation. Period hygiene products are frequently a sacrifice for women and girls who must choose between these necessities of life. Many women explained that financial hardship limited their ability to purchase menstrual products, forcing them to adopt coping strategies that they considered uncomfortable, unhygienic, or undignified. One participant stated:

“We live hand to mouth. If I spend money on pads, there will be less for food. I use cloth and wash it secretly, even if it’s uncomfortable or unhygienic.” *(IDI-5, 30 years, domestic worker)*

Another participant added:

“I cannot ask for pads every month. My father has little money, and my mother tells me to manage. Sometimes I use old cloth and hope it doesn’t show.” *(IDI-12, beauty parlor worker)*

NGO workers also emphasized how poverty interacts with stigma and social norms:

“Even when we provide pads or awareness sessions, many families cannot afford basic hygiene. Poverty amplifies the hardships and inequalities women face during menstruation. Social pressures make it worse.” *(KII-3, NGO Program Officer)*

## Discussion

This study examined how women and teenage girls residing in the informal settlements of Khulna Railway Slum, Bangladesh, manage menstrual health and hygiene amid environmental shortage, socio-cultural stigma, and economic disadvantage. The results indicate that menstruation is profoundly influenced by social and political factors, including gender, class, environment, and infrastructure, rather than being solely a biological phenomenon. Utilizing the Feminist Political Ecology (FPE) framework, the study contextualizes menstruation within broader power dynamics that govern access to material resources, influence bodily practices, and sustain inequalities through both overt and covert mechanisms of control [29,30]. The results indicate that access to water, sanitation, and menstrual waste management remains a persistent challenge for women and girls in informal settlements. Participants described difficulties managing menstruation due to inadequate sanitation facilities, limited privacy, and unsafe or unhygienic conditions. These findings correspond with studies undertaken in urban slums in Delhi, Nairobi, and Karachi [2,3,12], which demonstrate that insufficient WASH (water, sanitation, and hygiene) infrastructure, overcrowding, and lack of privacy substantially impede menstrual management [1,37,51]. Comparable findings have also been reported in Sub-Saharan African contexts, where women in informal settlements face significant barriers to managing menstruation safely due to inadequate facilities and concerns over dignity and safety [23,24,28].

Nonetheless, the Khulna environment introduces a unique aspect to this global dialogue. The coastal landscape, characterized by seasonal flooding, saline water intrusion, and high human density, exacerbates environmental vulnerability, significantly complicating menstrual management. Women in this study indicated utilizing rainwater collected in restricted containers and drying cloths in concealed, moist areas, techniques developed in response to environmental and spatial exigencies. These findings build upon previous research from Bangladesh [15] and Chennai [33], indicating that menstruation-related vulnerabilities are exacerbated in ecologically vulnerable and spatially limited metropolitan settings. From the perspective of FPE, the connection between environmental scarcity and physical experience demonstrates that women’s reproductive health is dependent on unequal access to natural resources, a dynamic that is both gendered and political [25,36].

Socio-cultural stigma revealed as a significant factor influencing menstruation experiences. Participants saw menstruation as a source of shame, concealment, and social marginalization, frequently refraining from routine tasks such as cooking, prayer, or attending school during their menstrual cycles. These accounts reflect research from India, Nepal, and Uganda [34,52,53], where menstruation is perceived as “impure” or “polluting,” resulting in physical and social isolation [20,54]. Research in Nepal has recorded severe instances of menstrual stigma, exemplified by ‘*chhaupadi’* [55] the isolation of menstruating women in designated huts [24,56], whereas in Bangladesh and India, limitations on diet, mobility, and engagement in religious activities continue [20,22].

Our research contributes to this body of literature by demonstrating that stigma is both culturally and spatially, as well as politically, rooted. Women in Khulna’s informal communities expressed the challenges of managing menstrual hygiene within cramped, single-room dwellings and under constant social scrutiny. They apprehended mockery when drying fabrics or acquiring goods from male vendors, illustrating how quotidian spatial configurations perpetuate gendered hierarchies and exacerbate shame. Global research highlights the impact of gendered spatiality on menstrual stigma. For instance, [33] in Delhi and [37] in Malawi demonstrate that women’s concerns about being observed or overheard during menstrual management limit their agency and mobility. This study emphasizes that stigma functions not just as a cultural taboo but also as a mechanism of power that regulates women’s bodies within inequitable material contexts [26,35]

Deficiencies in understanding menstruation exacerbate susceptibility. A significant number of adolescent females in this study encountered dread and perplexity during menarche, primarily attributable to intergenerational silence and inadequate school-based instruction. These findings align with those from Ethiopia [57], Kenya [58], and India [51], where inadequate menstrual education leads to ignorance, fear, and substandard hygiene practices. In Bangladesh, new research indicates that fewer than 50% of adolescent girls acquire accurate menstrual knowledge prior to menarche [4,15,18], often relying on friends or mothers who also lack expertise. Our findings demonstrate how this deficiency in information connects with socio-cultural stigma to establish cycles of silence, perpetuating gendered ignorance that constrains women’s autonomy.

Participants indicated a reduction in the prioritization of menstrual products within household budgets, frequently resorting to the reuse of outdated cloths or improvised materials. These experiences exemplify the widespread issue of “period poverty,” impacting millions of menstruators globally [1, 6). In Sub-Saharan Africa, as many as 65% of girls lack access to sanitary products [58], while in South Asia, cost remains a significant obstacle despite the availability of products [22,37]. This study emphasizes that in the Khulna environment, financial reliance on male household members constrains women’s capacity to make reproductive health decisions. The findings align with those of a study conducted in Uganda [23] and India [56]. The Feminist Political Ecology perspective clarifies that economic inequalities are not merely family concerns, but rather manifestations of systemic, gendered power dynamics that dictate access to resources and decision-making [25]. The convergence of environmental, social, and economic limitations has a significant impact on health and well-being. Women reported persistent infections, irritation, and discomfort resulting from unsanitary materials and hazardous washing conditions. International research substantiates the association between insufficient menstruation management and reproductive tract infections [22,54]. Furthermore, psychological effects such as shame, fear, anxiety, and limited engagement reflect results from multi-country studies indicating that menstruation stigma adversely affects mental well-being and social inclusion [1,58]. The repercussions for adolescent girls encompass educational outcomes, including school absenteeism resulting from insufficient facilities and embarrassment related to menstruation, which has been extensively documented in studies across six Sub-Saharan African countries [24], Nepal [55], and Bangladesh [17]. This study corroborates global concerns and highlights that environmental risks, such as floods, waterlogging, and hazardous public spaces, further impede education and mobility for girls in coastal informal communities.

### Policy implications

The findings indicate that menstrual health challenges in Khulna’s informal settlements are driven by inadequate WASH facilities, menstrual stigma, limited access to affordable menstrual products, weak institutional support, and persistent gender inequalities. Based on these findings, the following context-specific policy actions are recommended.

1. Improve gender-responsive WASH facilities in informal settlements

Participants consistently described the absence of private toilets, clean water, and safe disposal facilities as major barriers to managing menstruation safely and with dignity. Khulna City Corporation, in collaboration with the Department of Public Health Engineering (DPHE) and NGOs working in informal settlements, should prioritize upgrading communal sanitation facilities by ensuring lockable toilets, continuous water supply, handwashing stations, lighting, and covered bins for menstrual waste. Routine maintenance committees involving local women should also be established to ensure long-term functionality.

2. Integrate menstrual health education into schools and community programs

Many participants reported learning about menstruation only after menarche and described persistent myths, silence, and embarrassment within families and communities. The Ministry of Education, school authorities, and local NGOs should incorporate age-appropriate menstrual health education into existing school health programs. Community awareness sessions should also involve mothers, fathers, teachers, community leaders, and adolescent boys to normalize menstruation and reduce stigma beyond the classroom.

3. Increase access to affordable menstrual products

Women frequently reported using old cloth because sanitary pads were unaffordable or considered a low household priority. Local government, NGOs, and social enterprises should expand subsidized menstrual product distribution through community clinics, schools, and NGO outreach programs. Supporting women’s cooperatives and local entrepreneurs to produce affordable reusable menstrual products could improve access while generating livelihood opportunities for low-income women.

4. Create menstrual-friendly school environments

School-going girls described missing classes because of inadequate toilets, lack of water, and fear of menstrual leakage. Schools should ensure that girls have access to functional female toilets with water, disposal facilities, and emergency menstrual products. Teachers should receive practical training on menstrual health so they can provide confidential support and reduce stigma within the school environment.

5. Strengthen community-based support mechanisms

Participants consistently described menstruation as a topic that remained hidden within households, limiting opportunities to seek information or support. Community health workers, women’s groups, and local volunteers should facilitate regular menstrual health discussions within informal settlements using existing community platforms. These locally led initiatives can help address myths, improve knowledge, and encourage supportive household practices.

6. Mainstream menstrual health into urban poverty programs

The study demonstrates that menstrual health challenges are closely linked with poverty, insecure housing, and inadequate basic services. Rather than implementing stand-alone menstrual health projects, government agencies and development partners should integrate menstrual health into existing urban poverty reduction, slum upgrading, WASH, and primary healthcare programs operating in Khulna. Embedding menstrual health within ongoing urban development initiatives is likely to be more sustainable and cost-effective than creating separate programs.

### Strengths and limitations

This study provides valuable insights into the menstrual health and hygiene challenges faced by women and adolescent girls in the informal settlements of Khulna Railway Slum in Bangladesh. Rooted in feminist political ecology, it emphasizes how structural, environmental, and socio-cultural elements influence menstruation habits and disparities in access to safe hygiene facilities. The qualitative method, employing in-depth interviews, facilitated the acquisition of comprehensive narratives that encapsulate lived experiences frequently neglected in quantitative studies. Reflexivity and methodological triangulation enhanced the trustworthiness of the findings and reduced researcher bias. Utilizing a deductive qualitative technique may diminish flexibility in investigating emergent themes and heighten the possibility of confirmation bias, as researchers may prioritize data that corroborates established frameworks while disregarding unforeseen patterns. To address this, subsequent research should employ mixed-method approaches and utilize participatory tools to corroborate findings. Furthermore, the study’s context-specific characteristics and limited sample size may restrict the generalizability of the findings. This research significantly enhances our understanding of menstrual health disparities in urban informal settlements and provides essential guidance for gender-responsive MHH and public health initiatives.

## Conclusion

This study’s findings indicate that menstrual health and hygiene in Khulna’s informal settlements are significantly influenced by environmental shortage, socio-cultural stigma, and economic disadvantage. Women and adolescent girls confront these issues with adaptive techniques; nonetheless, their experiences are hindered by restricted access to potable water, insufficient sanitation, lack of privacy, and financial dependency. Menstruation is not solely a biological occurrence but a socially and politically influenced process that mirrors greater inequities in urban planning, domestic structures, and cultural conventions.

The present research underscores the significance of acknowledging menstrual health as an issue of dignity, equity, and social justice by exposing the lived realities of menstruators in precarious urban settings. Confronting these difficulties requires cohesive, contextually tailored initiatives that integrate enhancements in WASH infrastructure, menstrual education, product accessibility, and stigma alleviation. These initiatives can empower women, enhance their agency, and foster healthier, more inclusive urban communities where menstruation is managed safely, discreetly, and with dignity.
