# Supplementary material for: Unspoken cycles: A qualitative study exploring the lived experiences of women’s menstruation in the urban informal settlements of Bangladesh
Source: PLoS One. 2026 Jul 24;21(7):e0354260. doi: 10.1371/journal.pone.0354260 (PMC13399487; doi:10.1371/journal.pone.0354260)
Supplement: Attachment — Submitted filename: Review Comment_Manuscript title_Unspoken mestrual cycle.pdf [file pone.0354260.s001.pdf]

Manuscript title: Unspoken Cycles: A Qualitative Study Exploring the Lived Experiences of Women's Menstruation in the Urban Informal Settlements of Bangladesh.

Overall Assessment

This manuscript addresses an important and underexplored topic—menstrual health experiences among women and adolescent girls in informal urban settlements in Bangladesh. The qualitative approach and use of a Feminist Political Ecology framework are appropriate and have the potential to contribute meaningfully to the literature.

However, the manuscript currently has substantial methodological, structural, and analytical limitations, particularly in the Methods, Results, and Policy Recommendation sections. These issues affect the rigor, clarity, and internal consistency of the study.

Recommendation: Major Revision

1. Methods – Use of Citations and Conceptual Clarity
Location: Methods: Research Design section
The manuscript demonstrates several inconsistencies and weaknesses in the use of citations to justify methodological choices. Topic-specific qualitative studies (including authors' own prior work) are used to justify general methodological concepts, which is not appropriate.

Example:

- IDIs/KIIs justification supported by an applied study (Ref. 40)
- Qualitative approach justified using another applied study (Ref. 43)
-

These should be replaced with standard methodological references or removed. Although Braun & Clarke (2006) is appropriately cited, it is misapplied, with the introduction of non-standard terminology, "over-saturation in the findings," "feedback with analogous themes was regulated". These are not consistent with established qualitative analysis practices.

2. Methods – Missing Detail and Rigor (COREQ Issues)
Location: Methods: Sampling, Data Collection, Analysis
Several critical components required for qualitative rigor are missing or insufficiently described:

a. Sampling and Participants - No breakdown of adolescents vs adults and no explanation of age-based sampling strategy

b. Ethical procedures for adolescents (assent/parental consent) not described

c. Sampling Strategy- Combination of purposive and convenience sampling not justified

c. Recruitment- Unclear how participants were approached. No mention of refusals or recruitment flow.

d. Data Saturation described superficially (“no new themes emerged”)

e. No explanation of how saturation was assessed

f. Data Analysis- Inconsistency between deductive approach (stated earlier). Inductive + deductive approach (later introduced without explanation). No description of Codebook development, how codes evolved, or how discrepancies between coders were resolved.

g. Inter-coder Reliability- Two coders are mentioned, but no explanation of the agreement process, no description of consensus or validation.

h. Translation Process- Bengali to English translation described, but no explanation of validation or accuracy checks.

h. Trustworthiness- No discussion of Credibility, Dependability, Confirmability, Transferability. This is a major omission.

4. Reflexivity
Location: “Personality Statement” section
Reflexivity is acknowledged but not operationalized. No description of Reflexive journaling, team discussions, bias mitigation strategies

5. Results – Mixing with Discussion
Location: Results - multiple themes (e.g., Gendered Inequities)
The Results section includes interpretive and theoretical language that belongs in the Discussion: e.g., “surveillance and social visibility”, “confluence of economic, social, and gendered limitations”.

Results should focus on participant accounts and descriptive patterns. The Results also include literature comparisons, which should be moved to the discussion.

6. Results – Contextual Clarity
Location: Results: School-related findings
Discussion of school absenteeism lacks clarity. It is unclear whether barriers occur within or outside informal settlements. Given the study focus, findings should be clearly situated within the informal settlement context

7. Discussion – Unsupported Claims
Location: Discussion section
Some claims are not supported by the results: e.g., “dependence on pond water and rainwater collection”. No direct participant evidence supports this claim. This weakens traceability between data and interpretation

8. Policy Recommendations – Not Data-Driven (Critical Issue)
Location: Recommendations (especially 1, 4, 5)
Recommendations introduce climate adaptation, disaster management, and urban resilience. However, these themes are not present in the results. No participant data on: flooding, climate instability, disasters.

This represents a major coherence issue. Recommendations appear externally imposed rather than data-driven. Authors should either provide supporting evidence or revise recommendations to align with findings
