# Supplementary material for: Unspoken cycles: A qualitative study exploring the lived experiences of women’s menstruation in the urban informal settlements of Bangladesh
Source: PLoS One. 2026 Jul 24;21(7):e0354260. doi: 10.1371/journal.pone.0354260 (PMC13399487; doi:10.1371/journal.pone.0354260)
Supplement: Attachment — Submitted filename: Response to Reviewers-.docx [file pone.0354260.s002.docx]

**Detailed Response to Reviewers**

**Journal:** PLOS One

**Article type:** Original Research Article

**Manuscript title:** Unspoken Cycles: A Qualitative Study Exploring the Lived Experiences of Women’s Menstruation in the Urban Informal Settlements of Bangladesh

**Submission ID:** PONE-D-26-09182

**Comments from Reviewer-01**

*Your paper tackles a really important and often overlooked issue on how women and girls in urban informal settlements experience menstruation. The use of Feminist Political Ecology as a framework is a strong choice, and it helps connect individual struggles to broader structural and cultural forces. To make the manuscript stronger, you may consider tightening the abstract and introduction, so that the unique contribution of your study stands out more clearly. The methods section could use more detail on ethics approval and consent, especially since you worked with adolescents on a sensitive topic. In the findings, bringing in more direct quotes from participants would make the themes more vivid and ensure their voices remain central. The discussion section would benefit from a sharper focus on policy implications. Elaborate on how your findings could inform WASH infrastructure, education, and gender equity programs in Bangladesh.*

**Author Response:**

We sincerely thank the reviewer for the positive and constructive feedback. We are pleased that the reviewer recognizes the importance of the study and the relevance of the Feminist Political Ecology framework in understanding menstrual experiences within broader socio-structural contexts.

In response to the reviewer's suggestions, we have substantially revised the manuscript. First, the Abstract and Introduction sections have been refined to more clearly articulate the study's rationale, novelty, and contribution to the literature on menstrual health in urban informal settlements. Second, we have expanded the Methods section by providing additional details regarding ethical approval procedures, informed consent and assent processes, confidentiality measures, and protections implemented when conducting research with adolescent participants on a sensitive topic. (***Lines: 229-251***)

Third, we have incorporated additional verbatim quotations throughout the Findings section to better foreground participants' voices and strengthen the evidentiary basis of the identified themes. Finally, the Discussion section has been revised to provide a more focused consideration of policy and programmatic implications, particularly concerning WASH infrastructure improvements, menstrual health education, and gender-responsive interventions aimed at enhancing menstrual equity in urban informal settlements in Bangladesh.

**Comments from Reviewer-02**

*The manuscript addresses an important and underexplored topic- menstrual health experiences among women and adolescent girls in informal urban settlements in Bangladesh. The qualitative approach and use of a Feminist Political Ecology framework are appropriate and have the potential to contribute meaningfully to the literature. However, substantial revisions are required to improve methodological rigor, clarity, and alignment between findings and conclusions.*

***1. Methods – Use of Citations***

*The manuscript demonstrates several inconsistencies in the use of citations to justify methodological choices. Topic-specific qualitative studies (including the authors’ own prior work) are used to support general methodological claims (e.g., justification of IDIs/KIIs and qualitative approach), which is not appropriate. These should be replaced with standard methodological references or removed.*

*Additionally, while Braun and Clarke (2006) is appropriately cited, it is inconsistently applied, with the use of non-standard terminology (e.g., “over-saturation in the findings”), which is not supported by established qualitative frameworks.*

**Author Response:**

We sincerely thank the reviewer for this valuable observation. We agree that methodological decisions should be supported by established methodological literature rather than topic-specific empirical studies. In response, we carefully reviewed the Methods section and replaced several topic-specific references, including our own previous studies, with widely recognized methodological sources relevant to qualitative research design, in-depth interviews, key informant interviews, purposive sampling, data saturation, and thematic analysis. Where citations were not essential, they were removed to improve clarity and methodological precision.

We also revisited our description of the analytical process to ensure consistency with Braun and Clarke’s thematic analysis framework. The non-standard terminology previously used in the manuscript, including the phrase “over-saturation in the findings,” has been removed and replaced with terminology that is more consistent with established qualitative research standards. Furthermore, we refined the description of data saturation and thematic development to better reflect accepted qualitative methodological practices and to ensure conceptual alignment with Braun and Clarke’s approach.

***2. Methods – Missing Detail and Rigor***

*Several key components required for qualitative rigor (e.g., COREQ) are insufficiently described:*

*• Lack of clarity on sampling strategy and age distribution (adolescents vs adults)*

*• No justification for combining purposive and convenience sampling*

*• Weak description of data saturation*

*• No clear explanation of recruitment procedures*

*• Translation process not adequately described*

*• Inconsistency between deductive and inductive analysis approaches*

*• Lack of detail on inter-coder reliability and coding procedures*

*• Absence of discussion on trustworthiness (credibility, dependability, etc.)*

**Author Response:**

We sincerely thank the reviewer for this comprehensive and constructive feedback. We agree that the initial version of the manuscript did not adequately report several key elements of qualitative rigor as outlined in the COREQ guidelines. We have substantially revised the Methods section to address each of these concerns in detail. (***Lines: 229-324***)

**a. Sampling and Participant Characteristics:**

We have revised the Methods section to provide a clear breakdown of participants by age group, distinguishing between adolescent girls and adult women. We have also clarified the age-based sampling logic to show how participants were included to capture variation in lived menstrual experiences across life stages.

**b. Ethical Procedures for Adolescents:**

We have now explicitly detailed the ethical procedures related to adolescent participants, including parental/guardian consent and adolescent assent, along with additional safeguarding measures implemented during data collection.

**c. Sampling Strategy:**

We have clarified and justified the use of a combined purposive and limited convenience sampling strategy. The rationale for this approach has been added, emphasizing accessibility within informal settlements while ensuring variation in socio-demographic characteristics relevant to the study objectives.

**d. Recruitment Process and Non-Response:**

We have expanded the description of recruitment procedures, including how participants were approached through community entry points. We have also added information on recruitment flow, including whether any refusals occurred and how participation decisions were managed ethically and voluntarily.

**e. Data Saturation:**

We have revised the description of data saturation to provide a more methodologically grounded explanation. Instead of a general statement, we now describe how saturation was assessed through iterative data collection and analysis, specifically when no new codes or conceptual insights emerged across consecutive interviews.

**f. Data Analysis (Inductive and Deductive Approach):**

We have clarified the analytic approach by explicitly distinguishing between inductive and deductive coding processes. The revised manuscript now explains how an initial deductive framework informed early coding, followed by inductive refinement to allow new themes to emerge from the data. We have also added a detailed description of codebook development, iterative code refinement, and theme generation.

**g. Inter-coder Process:**

We have expanded the description of the two-coder process to include details on independent coding, comparison of coded transcripts, resolution of discrepancies through discussion, and consensus-based agreement. This has been added to strengthen transparency and analytic rigor.

**h. Translation Process:**

We have now elaborated the translation procedure, including forward translation from Bengali to English, cross-checking of translated transcripts, and validation through researcher review to ensure conceptual and linguistic accuracy.

**i. Trustworthiness Criteria:**

We have added a dedicated subsection on qualitative rigor, explicitly addressing credibility, dependability, confirmability, and transferability. This section outlines strategies such as prolonged engagement in the field, triangulation of perspectives (where applicable), reflexive discussions among researchers, audit trail maintenance, and thick description to enhance transferability.

***3. Reflexivity***

*The “Personality Statement” acknowledges reflexivity but does not explain how it was operationalized. Specific strategies (e.g., reflexive journaling, team discussions, bias mitigation) should be described.*

**Author Response:**

We thank the reviewer for this important observation. We agree that the original manuscript did not sufficiently operationalize reflexivity in a transparent and methodologically explicit manner. In response, we have revised the manuscript to clearly articulate the reflexive practices employed throughout the research process. (***Lines: 329-345***)

***4. Results Section***

*The Results section includes interpretive and theoretical language (e.g., “surveillance and social visibility”) that is more appropriate for the Discussion. Additionally, comparisons with existing literature appear in the Results, which should be moved to the Discussion.*

**Author Response:**

We sincerely thank the reviewer for this important and insightful observation. We agree that the original Results section included interpretive and theoretically framed language that blurred the distinction between results and discussion.

In response, we have thoroughly revised the Results section to improve structural and conceptual separation between empirical findings and interpretation. Specifically, we have removed theoretical and abstract framing from the Results and retained only descriptive presentation of themes supported by participants’ direct accounts.

***5. Results–Context Clarity***

*Some findings (e.g., school absenteeism) lack contextual clarity. It is unclear whether these challenges occur within or outside informal settlements. Stronger linkage to the study setting is needed.*

**Author Response:**

We thank the reviewer for this important clarification. We agree that the initial presentation of findings did not clearly specify the spatial and contextual link between informal settlement conditions and school-based experiences.

In response, we have revised the relevant section to explicitly situate school absenteeism within the lived realities of informal settlement residents. The revised text clarifies that educational barriers experienced by adolescent girls are shaped by conditions originating within informal settlements, including inadequate sanitation infrastructure, limited access to menstrual hygiene materials, and associated stigma, which subsequently affect school attendance and participation.

We have also refined the narrative to clearly distinguish between the setting of residence (informal settlements) and the setting of experience (schools), while emphasizing how structural disadvantages within settlements extend into educational spaces. This revision improves contextual clarity and strengthens alignment between the study setting and reported findings. (***Lines: 497-499 and 555-557***)

***6. Discussion – Unsupported Claims***

*Certain claims (e.g., dependence on pond water or rainwater) are not clearly supported by evidence in the Results. All interpretations should be grounded in participant data.*

**Author Response:**

We sincerely thank the reviewer for this important observation regarding the alignment between empirical findings and interpretive claims. We agree that the statement on reliance on pond water and rainwater collection was not sufficiently grounded in the presented results.

In response, we carefully re-examined the data and revised the Discussion section to ensure full consistency with the reported findings. Specifically, we have either removed or rephrased statements that were not directly supported by participant narratives or key informant accounts. Where relevant, we now ensure that all environmental and water-related claims are explicitly linked to corresponding empirical evidence from interviews, or otherwise reframed to reflect contextual background rather than direct findings. (***Lines: 588-597)***

***7. Policy Recommendations – Major Concern***

*Several recommendations (e.g., climate resilience, disaster management) are not supported by the study findings. There is no evidence from participant data indicating climate-related or disaster-related challenges. These recommendations appear externally introduced and should either be supported with data or revised to align with findings.*

**Author Response:**

We sincerely thank the reviewer for this important observation. We agree that policy recommendations should be closely grounded in the empirical findings of the study.

Upon revisiting the manuscript, we recognized that several recommendations concerning climate adaptation, disaster management, and urban resilience extended beyond the scope of the data presented in the Results section. As these themes were not explicitly raised by participants during interviews, we have removed or substantially revised these recommendations to ensure stronger alignment between findings and policy implications.

The revised recommendations now focus on issues directly identified by participants, including improvements in sanitation and WASH facilities, access to affordable menstrual hygiene materials, menstrual health education, school-based support systems, and community-level efforts to reduce menstrual stigma and gender-based barriers. These revisions strengthen the coherence of the manuscript and ensure that policy implications are firmly grounded in participants lived experiences and the evidence generated by the study. (***Lines: 661-703)***

We sincerely thank you for your constructive comments, which have helped us strengthen the manuscript. We look forward to your kind consideration of our revised submission.

Sincerely,

Md. Al-Mamun

Research Associate

BRAC Institute of Governance and Development (BIGD), BRAC University

Dhaka-1212, Bangladesh

Cell Phone Number: +8801797450408

Email: [md.mamun@bracu.ac.bd](mailto:md.mamun@bracu.ac.bd) or [mamunsiraji6576@gmail.com](mailto:mamunsiraji6576@gmail.com)

ORCID iD: <https://orcid.org/0000-0002-4133-757X>
