# Supplementary material for: Unspoken cycles: A qualitative study exploring the lived experiences of women’s menstruation in the urban informal settlements of Bangladesh
Source: PLoS One. 2026 Jul 24;21(7):e0354260. doi: 10.1371/journal.pone.0354260 (PMC13399487; doi:10.1371/journal.pone.0354260)
Supplement: Attachment — Submitted filename: Response to Reviewers-03-07-26.docx [file pone.0354260.s003.docx]

**Detailed Response to Reviewers**

**Journal:** PLOS One

**Article type:** Original Research Article

**Manuscript title:** Unspoken Cycles: A Qualitative Study Exploring the Lived Experiences of Women’s Menstruation in the Urban Informal Settlements of Bangladesh

**Submission ID:** PONE-D-26-09182R1

**Comments from Reviewer-01**

*Thank you for your revision. Here are additional areas for improvement.*

1. *Methodological Transparency: While you have added detail on sampling, recruitment, and translation, the reporting still feels uneven. For example, the justification for combining purposive and convenience sampling remains weak, and the explanation of data saturation is somewhat formulaic rather than demonstrating how saturation was assessed in practice.*

**Author Response:**

Thank you for this valuable suggestion. We have substantially strengthened the Sampling and Data Collection subsection. These revisions have been incorporated into the Methods section.

**(*Lines: 254-265, and 294-307)***

First, we now provide a clearer justification for combining purposive and convenience sampling. Purposive sampling was employed to intentionally recruit women and adolescent girls who met the predefined eligibility criteria and possessed direct experience of menstruation in informal settlements. Convenience sampling complemented this strategy because participant recruitment occurred within a resource-constrained urban slum where participant availability, household responsibilities, and willingness to participate varied considerably. Combining both approaches enabled us to access information-rich participants while accommodating practical constraints encountered during fieldwork.

Second, we have expanded the description of data saturation. Rather than simply stating that saturation was achieved, we now explain how saturation was assessed throughout the analytic process. After the fifteenth interview, no substantially new codes or conceptual categories emerged from the data. Three additional interviews were conducted to confirm that existing themes remained consistent and that no new insights were identified. This iterative process provided confidence that thematic saturation had been achieved.

2. *Reflexivity: The reflexive practices described (journaling, team discussions) are mentioned but not sufficiently illustrated with examples of how they influenced analytic decisions. This risks reflexivity being treated as a checklist rather than an integrated practice.*

**Author Response:** We appreciate this insightful observation. To address this concern, we have expanded the reflexivity section by providing concrete examples of how reflexive practice informed our analytical decisions throughout the study. **(*Lines: 342-366)***

Specifically, we explain that during the early stages of coding, researchers initially interpreted participants' descriptions of avoiding public spaces during menstruation primarily as consequences of inadequate sanitation infrastructure. However, through reflexive journaling and regular discussions among the research team, we recognized that these experiences also reflected internalized stigma, social expectations, and gendered surveillance within the community. Consequently, the coding framework was refined to distinguish structural constraints from socio-cultural mechanisms of exclusion. We also clarify that reflexive discussions helped challenge researchers' assumptions and ensured that interpretations remained grounded in participants' accounts rather than researchers' prior expectations.

3. *Findings Presentation: Although more participant quotations have been added, some themes still read as researcher-driven interpretations rather than grounded accounts. For instance, the framing of “social surveillance” and “visibility” feels imposed rather than emerging directly from participant narratives.*

**Author Response:** Thank you for this valuable observation. We carefully reviewed the Findings section to ensure that the analysis remains firmly grounded in participants lived experiences.

**(*Please have a look at Findings part now)***

In response to this comment, we revised sections where the narrative relied too heavily on researcher interpretation or abstract conceptual language. We strengthened the connection between participants' accounts and the analytical interpretation by incorporating additional verbatim quotations, reducing interpretive statements that were insufficiently supported by the data, and rewriting several transitional and summary paragraphs to foreground participants' own words and experiences.

Where participants described feelings of embarrassment, fear of being judged, the need to conceal menstruation, restrictions on mobility, or difficulties accessing menstrual products, these accounts are now presented first, with analytical interpretation introduced only after sufficient empirical evidence has been established. We also revised several descriptive passages to use participant-centred language (e.g., "participants described...", "women reported...", and "their accounts suggest...") rather than researcher-driven assertions.

4. *Policy Implications: The discussion now avoids unsupported claims (e.g., climate resilience), but the recommendations remain somewhat generic. Stronger linkage between specific findings (e.g., inadequate WASH facilities, stigma in schools) and actionable interventions would make the policy relevance sharper.*

**Author Response:** Thank you for this valuable suggestion. We have substantially revised the Policy Implications section to establish a clearer and more explicit connection between our empirical findings and the recommended interventions. Rather than presenting broad recommendations, each policy implication is now directly informed by specific findings of the study. **(*Lines: 682-727)***

For example, participants' experiences of inadequate access to private toilets, clean water, and menstrual waste disposal facilities are now directly linked to recommendations for strengthening gender-responsive WASH infrastructure in informal settlements and schools. Similarly, findings related to menstrual stigma, household silence, misinformation, and school absenteeism have been connected to specific interventions, including integrating comprehensive menstrual health education into school curricula, teacher sensitization, community awareness programs involving parents and men, and the establishment of menstrual-friendly school environments. Recommendations addressing the affordability of menstrual products are now explicitly based on participants' accounts of financial hardship and reliance on reusable cloth, leading to proposals for subsidized menstrual products, community-based distribution mechanisms, and support for locally produced reusable menstrual products. We also strengthened the recommendations by identifying relevant implementation stakeholders, including local government authorities, educational institutions, NGOs, community health workers, and development partners, making the proposed interventions more practical and contextually relevant for informal settlements in Bangladesh.

We sincerely thank you for your constructive comments, which have helped us strengthen the manuscript. We look forward to your kind consideration of our revised submission.

Sincerely,

Md. Al-Mamun

Research Associate

BRAC Institute of Governance and Development (BIGD), BRAC University

Dhaka-1212, Bangladesh

Cell Phone Number: +8801797450408

Email: [md.mamun@bracu.ac.bd](mailto:md.mamun@bracu.ac.bd) or [mamunsiraji6576@gmail.com](mailto:mamunsiraji6576@gmail.com)

ORCID iD: <https://orcid.org/0000-0002-4133-757X>
