# Supplementary material for: Unspoken cycles: A qualitative study exploring the lived experiences of women’s menstruation in the urban informal settlements of Bangladesh
Source: PLoS One. 2026 Jul 24;21(7):e0354260. doi: 10.1371/journal.pone.0354260 (PMC13399487; doi:10.1371/journal.pone.0354260)
Supplement: Peer review history

# Peer review: Decision Letter 0

**DOI:** 10.1371/journal.pone.0354260.r001

**Authors:** Alison Parker

20 Apr 2026

PONE-D-26-09182

Unspoken Cycles: A Qualitative Study Exploring the Lived Experiences of Women’s Menstruation in the Urban Informal Settlements of Bangladesh

PLOS One

Dear Dr. Al-Mamun,

Thank you for submitting your manuscript to PLOS ONE. After careful consideration, we feel that it has merit but does not fully meet PLOS ONE’s publication criteria as it currently stands. Therefore, we invite you to submit a revised version of the manuscript that addresses the points raised during the review process.

There are some substantive comments to address here particularly on the methods section.

Please submit your revised manuscript by Jun 04 2026 11:59PM. If you will need more time than this to complete your revisions, please reply to this message or contact the journal office at plosone@plos.org. When you're ready to submit your revision, log on to https://www.editorialmanager.com/pone/ and select the 'Submissions Needing Revision' folder to locate your manuscript file.

Please include the following items when submitting your revised manuscript:

- A letter that responds to each point raised by the academic editor and reviewer(s). You should upload this letter as a separate file labeled 'Response to Reviewers'.
- A marked-up copy of your manuscript that highlights changes made to the original version. You should upload this as a separate file labeled 'Revised Manuscript with Track Changes'.
- An unmarked version of your revised paper without tracked changes. You should upload this as a separate file labeled 'Manuscript'.

If you would like to make changes to your financial disclosure, please include your updated statement in your cover letter. Guidelines for resubmitting your figure files are available below the reviewer comments at the end of this letter.

If applicable, we recommend that you deposit your laboratory protocols in protocols.io to enhance the reproducibility of your results. Protocols.io assigns your protocol its own identifier (DOI) so that it can be cited independently in the future. For instructions see: https://journals.plos.org/plosone/s/submission-guidelines#loc-laboratory-protocols. Additionally, PLOS ONE offers an option for publishing peer-reviewed Lab Protocol articles, which describe protocols hosted on protocols.io. Read more information on sharing protocols at https://plos.org/protocols?utm_medium=editorial-email&utm_source=authorletters&utm_campaign=protocols.

As the corresponding author, your ORCID iD is verified in the submission system and will appear in the published article. PLOS supports the use of ORCID, and we encourage all coauthors to register for an ORCID iD and use it as well. Please encourage your coauthors to verify their ORCID iD within the submission system before final acceptance, as unverified ORCID iDs will not appear in the published article. *Only*  the individual author can complete the verification step; PLOS staff *cannot*  verify ORCID iDs on behalf of authors.

We look forward to receiving your revised manuscript.

Kind regards,

Alison Parker

Academic Editor

PLOS One

Journal Requirements:

When submitting your revision, we need you to address these additional requirements.

1. Please ensure that your manuscript meets PLOS ONE's style requirements, including those for file naming. The PLOS ONE style templates can be found at

https://journals.plos.org/plosone/s/file?id=wjVg/PLOSOne_formatting_sample_main_body.pdf and https://journals.plos.org/plosone/s/file?id=ba62/PLOSOne_formatting_sample_title_authors_affiliations.pdf

2. Please include a complete copy of PLOS’ questionnaire on inclusivity in global research in your revised manuscript. Our policy for research in this area aims to improve transparency in the reporting of research performed outside of researchers’ own country or community. The policy applies to researchers who have travelled to a different country to conduct research, research with Indigenous populations or their lands, and research on cultural artefacts. The questionnaire can also be requested at the journal’s discretion for any other submissions, even if these conditions are not met.  Please find more information on the policy and a link to download a blank copy of the questionnaire here: https://journals.plos.org/plosone/s/best-practices-in-research-reporting. Please upload a completed version of your questionnaire as Supporting Information when you resubmit your manuscript.

3. Please provide a complete Data Availability Statement in the submission form, ensuring you include all necessary access information or a reason for why you are unable to make your data freely accessible. If your research concerns only data provided within your submission, please write "All data are in the manuscript and/or supporting information files" as your Data Availability Statement.

4. When completing the data availability statement of the submission form, you indicated that you will make your data available on acceptance. We strongly recommend all authors decide on a data sharing plan before acceptance, as the process can be lengthy and hold up publication timelines. Please note that, though access restrictions are acceptable now, your entire data will need to be made freely accessible if your manuscript is accepted for publication. This policy applies to all data except where public deposition would breach compliance with the protocol approved by your research ethics board. If you are unable to adhere to our open data policy, please kindly revise your statement to explain your reasoning and we will seek the editor's input on an exemption. Please be assured that, once you have provided your new statement, the assessment of your exemption will not hold up the peer review process.

5. Thank you for stating the following in your Competing Interests section:

N/A

Please complete your Competing Interests on the online submission form to state any Competing Interests. If you have no competing interests, please state "The authors have declared that no competing interests exist.", as detailed online in our guide for authors at http://journals.plos.org/plosone/s/submit-now

This information should be included in your cover letter; we will change the online submission form on your behalf.

6. Please include a separate caption for each figure in your manuscript.

7. We note that Figure 1 in your submission contain copyrighted images. All PLOS content is published under the Creative Commons Attribution License (CC BY 4.0), which means that the manuscript, images, and Supporting Information files will be freely available online, and any third party is permitted to access, download, copy, distribute, and use these materials in any way, even commercially, with proper attribution. For more information, see our copyright guidelines: http://journals.plos.org/plosone/s/licenses-and-copyright.

We require you to either (1) present written permission from the copyright holder to publish these figures specifically under the CC BY 4.0 license, or (2) remove the figures from your submission:

a. You may seek permission from the original copyright holder of Figure 1 to publish the content specifically under the CC BY 4.0 license.

We recommend that you contact the original copyright holder with the Content Permission Form (http://journals.plos.org/plosone/s/file?id=7c09/content-permission-form.pdf) and the following text:

“I request permission for the open-access journal PLOS ONE to publish XXX under the Creative Commons Attribution License (CCAL) CC BY 4.0 (http://creativecommons.org/licenses/by/4.0/). Please be aware that this license allows unrestricted use and distribution, even commercially, by third parties. Please reply and provide explicit written permission to publish XXX under a CC BY license and complete the attached form.”

Please upload the completed Content Permission Form or other proof of granted permissions as an "Other" file with your submission.

In the figure caption of the copyrighted figure, please include the following text: “Reprinted from [ref] under a CC BY license, with permission from [name of publisher], original copyright [original copyright year].”

b. If you are unable to obtain permission from the original copyright holder to publish these figures under the CC BY 4.0 license or if the copyright holder’s requirements are incompatible with the CC BY 4.0 license, please either i) remove the figure or ii) supply a replacement figure that complies with the CC BY 4.0 license. Please check copyright information on all replacement figures and update the figure caption with source information. If applicable, please specify in the figure caption text when a figure is similar but not identical to the original image and is therefore for illustrative purposes only.

8. We note that Figure 2 in your submission contain [map/satellite] images which may be copyrighted. All PLOS content is published under the Creative Commons Attribution License (CC BY 4.0), which means that the manuscript, images, and Supporting Information files will be freely available online, and any third party is permitted to access, download, copy, distribute, and use these materials in any way, even commercially, with proper attribution. For these reasons, we cannot publish previously copyrighted maps or satellite images created using proprietary data, such as Google software (Google Maps, Street View, and Earth). For more information, see our copyright guidelines: http://journals.plos.org/plosone/s/licenses-and-copyright.

We require you to either (1) present written permission from the copyright holder to publish these figures specifically under the CC BY 4.0 license, or (2) remove the figures from your submission:

a. You may seek permission from the original copyright holder of Figure 2 to publish the content specifically under the CC BY 4.0 license.

We recommend that you contact the original copyright holder with the Content Permission Form (http://journals.plos.org/plosone/s/file?id=7c09/content-permission-form.pdf) and the following text:

“I request permission for the open-access journal PLOS ONE to publish XXX under the Creative Commons Attribution License (CCAL) CC BY 4.0 (http://creativecommons.org/licenses/by/4.0/). Please be aware that this license allows unrestricted use and distribution, even commercially, by third parties. Please reply and provide explicit written permission to publish XXX under a CC BY license and complete the attached form.”

Please upload the completed Content Permission Form or other proof of granted permissions as an "Other" file with your submission.

In the figure caption of the copyrighted figure, please include the following text: “Reprinted from [ref] under a CC BY license, with permission from [name of publisher], original copyright [original copyright year].”

b. If you are unable to obtain permission from the original copyright holder to publish these figures under the CC BY 4.0 license or if the copyright holder’s requirements are incompatible with the CC BY 4.0 license, please either i) remove the figure or ii) supply a replacement figure that complies with the CC BY 4.0 license. Please check copyright information on all replacement figures and update the figure caption with source information. If applicable, please specify in the figure caption text when a figure is similar but not identical to the original image and is therefore for illustrative purposes only.

The following resources for replacing copyrighted map figures may be helpful:

USGS National Map Viewer (public domain): http://viewer.nationalmap.gov/viewer/

The Gateway to Astronaut Photography of Earth (public domain): http://eol.jsc.nasa.gov/sseop/clickmap/

Maps at the CIA (public domain): https://www.cia.gov/library/publications/the-world-factbook/index.html and https://www.cia.gov/library/publications/cia-maps-publications/index.html

NASA Earth Observatory (public domain): http://earthobservatory.nasa.gov/

Landsat: http://landsat.visibleearth.nasa.gov/

USGS EROS (Earth Resources Observatory and Science (EROS) Center) (public domain): http://eros.usgs.gov/#

Natural Earth (public domain): http://www.naturalearthdata.com/

9. If the reviewer comments include a recommendation to cite specific previously published works, please review and evaluate these publications to determine whether they are relevant and should be cited. There is no requirement to cite these works unless the editor has indicated otherwise.

[Note: HTML markup is below. Please do not edit.]

Reviewers' comments:

Reviewer's Responses to Questions

**Comments to the Author**

1. Is the manuscript technically sound, and do the data support the conclusions?

The manuscript must describe a technically sound piece of scientific research with data that supports the conclusions. Experiments must have been conducted rigorously, with appropriate controls, replication, and sample sizes. The conclusions must be drawn appropriately based on the data presented.

Reviewer #1: Yes

Reviewer #2: No

**********

2. Has the statistical analysis been performed appropriately and rigorously?

Reviewer #1: N/A

Reviewer #2: N/A

**********

3. Have the authors made all data underlying the findings in their manuscript fully available?

The PLOS Data policy requires authors to make all data underlying the findings described in their manuscript fully available without restriction, with rare exception (please refer to the Data Availability Statement in the manuscript PDF file). The data should be provided as part of the manuscript or its supporting information, or deposited to a public repository. For example, in addition to summary statistics, the data points behind means, medians and variance measures should be available. If there are restrictions on publicly sharing data—e.g. participant privacy or use of data from a third party—those must be specified.

Reviewer #1: Yes

Reviewer #2: No

**********

4. Is the manuscript presented in an intelligible fashion and written in standard English?

PLOS ONE does not copyedit accepted manuscripts, so the language in submitted articles must be clear, correct, and unambiguous. Any typographical or grammatical errors should be corrected at revision, so please note any specific errors here.

Reviewer #1: Yes

Reviewer #2: No

**********

5. Review Comments to the Author

Please use the space provided to explain your answers to the questions above. You may also include additional comments for the author, including concerns about dual publication, research ethics, or publication ethics. (Please upload your review as an attachment if it exceeds 20,000 characters)

Reviewer #1: Your paper tackles a really important and often overlooked issue on how women and girls in urban informal settlements experience menstruation. The use of Feminist Politica Ecology as a framework is a strong choice, and it helps connect individual struggles to broader structural and cultural forces. To make the manuscript stronger, you may consider tightening the abstract and introduction, so that the unique contribution of your study stands out more clearly. The methods section could use more detail on ethics approval and consent, especially since you worked with adolescents on a sensitive topic. In the findings, bringing in more direct quotes from participants would make the themes more vivid and ensure their voices remain central. The discussion section would benefit from a sharper focus on policy implications. Elaborate on how your findings could inform WASH infrastructure, education, and gender equity programs in Bangladesh.

Reviewer #2: Review Comments to the Author

The manuscript addresses an important and underexplored topic- menstrual health experiences among women and adolescent girls in informal urban settlements in Bangladesh. The qualitative approach and use of a Feminist Political Ecology framework are appropriate and have the potential to contribute meaningfully to the literature. However, substantial revisions are required to improve methodological rigor, clarity, and alignment between findings and conclusions.

Major Comments

1. Methods – Use of Citations

The manuscript demonstrates several inconsistencies in the use of citations to justify methodological choices. Topic-specific qualitative studies (including the authors’ own prior work) are used to support general methodological claims (e.g., justification of IDIs/KIIs and qualitative approach), which is not appropriate. These should be replaced with standard methodological references or removed.

Additionally, while Braun and Clarke (2006) is appropriately cited, it is inconsistently applied, with the use of non-standard terminology (e.g., “over-saturation in the findings”), which is not supported by established qualitative frameworks.

2. Methods – Missing Detail and Rigor

Several key components required for qualitative rigor (e.g., COREQ) are insufficiently described:

• Lack of clarity on sampling strategy and age distribution (adolescents vs adults)

• No justification for combining purposive and convenience sampling

• Weak description of data saturation

• No clear explanation of recruitment procedures

• Translation process not adequately described

• Inconsistency between deductive and inductive analysis approaches

• Lack of detail on inter-coder reliability and coding procedures

• Absence of discussion on trustworthiness (credibility, dependability, etc.)

3. Reflexivity

The “Personality Statement” acknowledges reflexivity but does not explain how it was operationalized. Specific strategies (e.g., reflexive journaling, team discussions, bias mitigation) should be described.

4. Results Section

The Results section includes interpretive and theoretical language (e.g., “surveillance and social visibility”) that is more appropriate for the Discussion. Additionally, comparisons with existing literature appear in the Results, which should be moved to the Discussion.

5. Results–Context Clarity

Some findings (e.g., school absenteeism) lack contextual clarity. It is unclear whether these challenges occur within or outside informal settlements. Stronger linkage to the study setting is needed.

6. Discussion – Unsupported Claims

Certain claims (e.g., dependence on pond water or rainwater) are not clearly supported by evidence in the Results. All interpretations should be grounded in participant data.

7. Policy Recommendations – Major Concern

Several recommendations (e.g., climate resilience, disaster management) are not supported by the study findings. There is no evidence from participant data indicating climate-related or disaster-related challenges. These recommendations appear externally introduced and should either be supported with data or revised to align with findings.

Minor Comments

• Multiple grammatical and phrasing issues throughout the manuscript

• Repetition and verbosity in some sections

• Awkward sentence structures in Methods

Recommendation

Major Revision

**********

6. PLOS authors have the option to publish the peer review history of their article (what does this mean?). If published, this will include your full peer review and any attached files.

If you choose “no”, your identity will remain anonymous but your review may still be made public.

**Do you want your identity to be public for this peer review?**  For information about this choice, including consent withdrawal, please see our Privacy Policy.

Reviewer #1: No

Reviewer #2: No

**********

[NOTE: If reviewer comments were submitted as an attachment file, they will be attached to this email and accessible via the submission site. Please log into your account, locate the manuscript record, and check for the action link "View Attachments". If this link does not appear, there are no attachment files.]

To ensure your figures meet our technical requirements, please review our figure guidelines: https://journals.plos.org/plosone/s/figures

You may also use PLOS’s free figure tool, NAAS, to help you prepare publication quality figures: https://journals.plos.org/plosone/s/figures#loc-tools-for-figure-preparation.

NAAS will assess whether your figures meet our technical requirements by comparing each figure against our figure specifications.

Attachment Submitted filename: Review Comment_Manuscript title_Unspoken mestrual cycle.pdf

# Author comment: Author response to Decision Letter 1

**DOI:** 10.1371/journal.pone.0354260.r002

15 Jun 2026

Manuscript ID: PONE-D-26-09182

Title: Unspoken Cycles: A Qualitative Study Exploring the Lived Experiences of Women’s Menstruation in the Urban Informal Settlements of Bangladesh

Response to Academic Editor

We sincerely thank the Academic Editor for the opportunity to revise and resubmit our manuscript. We appreciate the careful evaluation and constructive feedback. We have thoroughly revised the manuscript in line with the reviewer comments, with particular attention to methodological rigor, clarity, and alignment between findings and conclusions. All revisions have strengthened the overall structure, reporting quality, and policy relevance of the study.

Comments from Reviewer-01

Your paper tackles a really important and often overlooked issue on how women and girls in urban informal settlements experience menstruation. The use of Feminist Political Ecology as a framework is a strong choice, and it helps connect individual struggles to broader structural and cultural forces. To make the manuscript stronger, you may consider tightening the abstract and introduction, so that the unique contribution of your study stands out more clearly. The methods section could use more detail on ethics approval and consent, especially since you worked with adolescents on a sensitive topic. In the findings, bringing in more direct quotes from participants would make the themes more vivid and ensure their voices remain central. The discussion section would benefit from a sharper focus on policy implications. Elaborate on how your findings could inform WASH infrastructure, education, and gender equity programs in Bangladesh.

Author Response:

We sincerely thank the reviewer for the positive and constructive feedback. We are pleased that the reviewer recognizes the importance of the study and the relevance of the Feminist Political Ecology framework in understanding menstrual experiences within broader socio-structural contexts.

In response to the reviewer's suggestions, we have substantially revised the manuscript. First, the Abstract and Introduction sections have been refined to more clearly articulate the study's rationale, novelty, and contribution to the literature on menstrual health in urban informal settlements. Second, we have expanded the Methods section by providing additional details regarding ethical approval procedures, informed consent and assent processes, confidentiality measures, and protections implemented when conducting research with adolescent participants on a sensitive topic. (Lines: 229-251)

Third, we have incorporated additional verbatim quotations throughout the Findings section to better foreground participants' voices and strengthen the evidentiary basis of the identified themes. Finally, the Discussion section has been revised to provide a more focused consideration of policy and programmatic implications, particularly concerning WASH infrastructure improvements, menstrual health education, and gender-responsive interventions aimed at enhancing menstrual equity in urban informal settlements in Bangladesh.

Comments from Reviewer-02

The manuscript addresses an important and underexplored topic- menstrual health experiences among women and adolescent girls in informal urban settlements in Bangladesh. The qualitative approach and use of a Feminist Political Ecology framework are appropriate and have the potential to contribute meaningfully to the literature. However, substantial revisions are required to improve methodological rigor, clarity, and alignment between findings and conclusions.

1. Methods – Use of Citations

The manuscript demonstrates several inconsistencies in the use of citations to justify methodological choices. Topic-specific qualitative studies (including the authors’ own prior work) are used to support general methodological claims (e.g., justification of IDIs/KIIs and qualitative approach), which is not appropriate. These should be replaced with standard methodological references or removed.

Additionally, while Braun and Clarke (2006) is appropriately cited, it is inconsistently applied, with the use of non-standard terminology (e.g., “over-saturation in the findings”), which is not supported by established qualitative frameworks.

Author Response:

We sincerely thank the reviewer for this valuable observation. We agree that methodological decisions should be supported by established methodological literature rather than topic-specific empirical studies. In response, we carefully reviewed the Methods section and replaced several topic-specific references, including our own previous studies, with widely recognized methodological sources relevant to qualitative research design, in-depth interviews, key informant interviews, purposive sampling, data saturation, and thematic analysis. Where citations were not essential, they were removed to improve clarity and methodological precision.

We also revisited our description of the analytical process to ensure consistency with Braun and Clarke’s thematic analysis framework. The non-standard terminology previously used in the manuscript, including the phrase “over-saturation in the findings,” has been removed and replaced with terminology that is more consistent with established qualitative research standards. Furthermore, we refined the description of data saturation and thematic development to better reflect accepted qualitative methodological practices and to ensure conceptual alignment with Braun and Clarke’s approach.

2. Methods – Missing Detail and Rigor

Several key components required for qualitative rigor (e.g., COREQ) are insufficiently described:

• Lack of clarity on sampling strategy and age distribution (adolescents vs adults)

• No justification for combining purposive and convenience sampling

• Weak description of data saturation

• No clear explanation of recruitment procedures

• Translation process not adequately described

• Inconsistency between deductive and inductive analysis approaches

• Lack of detail on inter-coder reliability and coding procedures

• Absence of discussion on trustworthiness (credibility, dependability, etc.)

Author Response:

We sincerely thank the reviewer for this comprehensive and constructive feedback. We agree that the initial version of the manuscript did not adequately report several key elements of qualitative rigor as outlined in the COREQ guidelines. We have substantially revised the Methods section to address each of these concerns in detail. (Lines: 229-324)

a. Sampling and Participant Characteristics:

We have revised the Methods section to provide a clear breakdown of participants by age group, distinguishing between adolescent girls and adult women. We have also clarified the age-based sampling logic to show how participants were included to capture variation in lived menstrual experiences across life stages.

b. Ethical Procedures for Adolescents:

We have now explicitly detailed the ethical procedures related to adolescent participants, including parental/guardian consent and adolescent assent, along with additional safeguarding measures implemented during data collection.

c. Sampling Strategy:

We have clarified and justified the use of a combined purposive and limited convenience sampling strategy. The rationale for this approach has been added, emphasizing accessibility within informal settlements while ensuring variation in socio-demographic characteristics relevant to the study objectives.

d. Recruitment Process and Non-Response:

We have expanded the description of recruitment procedures, including how participants were approached through community entry points. We have also added information on recruitment flow, including whether any refusals occurred and how participation decisions were managed ethically and voluntarily.

e. Data Saturation:

We have revised the description of data saturation to provide a more methodologically grounded explanation. Instead of a general statement, we now describe how saturation was assessed through iterative data collection and analysis, specifically when no new codes or conceptual insights emerged across consecutive interviews.

f. Data Analysis (Inductive and Deductive Approach):

We have clarified the analytic approach by explicitly distinguishing between inductive and deductive coding processes. The revised manuscript now explains how an initial deductive framework informed early coding, followed by inductive refinement to allow new themes to emerge from the data. We have also added a detailed description of codebook development, iterative code refinement, and theme generation.

g. Inter-coder Process:

We have expanded the description of the two-coder process to include details on independent coding, comparison of coded transcripts, resolution of discrepancies through discussion, and consensus-based agreement. This has been added to strengthen transparency and analytic rigor.

h. Translation Process:

We have now elaborated the translation procedure, including forward translation from Bengali to English, cross-checking of translated transcripts, and validation through researcher review to ensure conceptual and linguistic accuracy.

i. Trustworthiness Criteria:

We have added a dedicated subsection on qualitative rigor, explicitly addressing credibility, dependability, confirmability, and transferability. This section outlines strategies such as prolonged engagement in the field, triangulation of perspectives (where applicable), reflexive discussions among researchers, audit trail maintenance, and thick description to enhance transferability.

3. Reflexivity

The “Personality Statement” acknowledges reflexivity but does not explain how it was operationalized. Specific strategies (e.g., reflexive journaling, team discussions, bias mitigation) should be described.

Author Response:

We thank the reviewer for this important observation. We agree that the original manuscript did not sufficiently operationalize reflexivity in a transparent and methodologically explicit manner. In response, we have revised the manuscript to clearly articulate the reflexive practices employed throughout the research process. (Lines: 329-345)

4. Results Section

The Results section includes interpretive and theoretical language (e.g., “surveillance and social visibility”) that is more appropriate for the Discussion. Additionally, comparisons with existing literature appear in the Results, which should be moved to the Discussion.

Author Response:

We sincerely thank the reviewer for this important and insightful observation. We agree that the original Results section included interpretive and theoretically framed language that blurred the distinction between results and discussion.

In response, we have thoroughly revised the Results section to improve structural and conceptual separation between empirical findings and interpretation. Specifically, we have removed theoretical and abstract framing from the Results and retained only descriptive presentation of themes supported by participants’ direct accounts.

5. Results–Context Clarity

Some findings (e.g., school absenteeism) lack contextual clarity. It is unclear whether these challenges occur within or outside informal settlements. Stronger linkage to the study setting is needed.

Author Response:

We thank the reviewer for this important clarification. We agree that the initial presentation of findings did not clearly specify the spatial and contextual link between informal settlement conditions and school-based experiences.

In response, we have revised the relevant section to explicitly situate school absenteeism within the lived realities of informal settlement residents. The revised text clarifies that educational barriers experienced by adolescent girls are shaped by conditions originating within informal settlements, including inadequate sanitation infrastructure, limited access to menstrual hygiene materials, and associated stigma, which subsequently affect school attendance and participation.

We have also refined the narrative to clearly distinguish between the setting of residence (informal settlements) and the setting of experience (schools), while emphasizing how structural disadvantages within settlements extend into educational spaces. This revision improves contextual clarity and strengthens alignment between the study setting and reported findings. (Lines: 497-499 and 555-557)

6. Discussion – Unsupported Claims

Certain claims (e.g., dependence on pond water or rainwater) are not clearly supported by evidence in the Results. All interpretations should be grounded in participant data.

Author Response:

We sincerely thank the reviewer for this important observation regarding the alignment between empirical findings and interpretive claims. We agree that the statement on reliance on pond water and rainwater collection was not sufficiently grounded in the presented results.

In response, we carefully re-examined the data and revised the Discussion section to ensure full consistency with the reported findings. Specifically, we have either removed or rephrased statements that were not directly supported by participant narratives or key informant accounts. Where relevant, we now ensure that all environmental and water-related claims are explicitly linked to corresponding empirical evidence from interviews, or otherwise reframed to reflect contextual background rather than direct findings. (Lines: 588-597)

7. Policy Recommendations – Major Concern

Several recommendations (e.g., climate resilience, disaster management) are not supported by the study findings. There is no evidence from participant data indicating climate-related or disaster-related challenges. These recommendations appear externally introduced and should either be supported with data or revised to align with findings.

Author Response:

We sincerely thank the reviewer for this important observation. We agree that policy recommendations should be closely grounded in the empirical findings of the study.

Upon revisiting the manuscript, we recognized that several recommendations concerning climate adaptation, disaster management, and urban resilience extended beyond the scope of the data presented in the Results section. As these themes were not explicitly raised by participants during interviews, we have removed or substantially revised these recommendations to ensure stronger alignment between findings and policy implications.

The revised recommendations now focus on issues directly identified by participants, including improvements in sanitation and WASH facilities, access to affordable menstrual hygiene materials, menstrual health education, school-based support systems, and community-level efforts to reduce menstrual stigma and gender-based barriers. These revisions strengthen the coherence of the manuscript and ensure that policy implications are firmly grounded in participants lived experiences and the evidence generated by the study. (Lines: 661-703)

We sincerely thank you for your constructive comments, which have helped us strengthen the manuscript. We look forward to your kind consideration of our revised submission.

Sincerely,

Md. Al-Mamun

Research Associate

BRAC Institute of Governance and Development (BIGD), BRAC University

Dhaka-1212, Bangladesh

Cell Phone Number: +8801797450408

Email: md.mamun@bracu.ac.bd or mamunsiraji6576@gmail.com

ORCID iD: https://orcid.org/0000-0002-4133-757X

Attachment Submitted filename: Response to Reviewers-.docx

# Peer review: Decision Letter 1

**DOI:** 10.1371/journal.pone.0354260.r003

**Authors:** Alison Parker

30 Jun 2026

PONE-D-26-09182R1

Unspoken Cycles: A Qualitative Study Exploring the Lived Experiences of Women’s Menstruation in the Urban Informal Settlements of Bangladesh

PLOS One

Dear Dr. Al-Mamun,

Thank you for submitting your manuscript to PLOS ONE. After careful consideration, we feel that it has merit but does not fully meet PLOS ONE’s publication criteria as it currently stands. Therefore, we invite you to submit a revised version of the manuscript that addresses the points raised during the review process.

Please submit your revised manuscript by Aug 14 2026 11:59PM. If you will need more time than this to complete your revisions, please reply to this message or contact the journal office at plosone@plos.org. When you're ready to submit your revision, log on to https://www.editorialmanager.com/pone/ and select the 'Submissions Needing Revision' folder to locate your manuscript file.

Please include the following items when submitting your revised manuscript:

- A letter that responds to each point raised by the academic editor and reviewer(s). You should upload this letter as a separate file labeled 'Response to Reviewers'.
- A marked-up copy of your manuscript that highlights changes made to the original version. You should upload this as a separate file labeled 'Revised Manuscript with Track Changes'.
- An unmarked version of your revised paper without tracked changes. You should upload this as a separate file labeled 'Manuscript'.

If you would like to make changes to your financial disclosure, please include your updated statement in your cover letter. Guidelines for resubmitting your figure files are available below the reviewer comments at the end of this letter.

If applicable, we recommend that you deposit your laboratory protocols in protocols.io to enhance the reproducibility of your results. Protocols.io assigns your protocol its own identifier (DOI) so that it can be cited independently in the future. For instructions see: https://journals.plos.org/plosone/s/submission-guidelines#loc-laboratory-protocols. Additionally, PLOS ONE offers an option for publishing peer-reviewed Lab Protocol articles, which describe protocols hosted on protocols.io. Read more information on sharing protocols at https://plos.org/protocols?utm_medium=editorial-email&utm_source=authorletters&utm_campaign=protocols.

As the corresponding author, your ORCID iD is verified in the submission system and will appear in the published article. PLOS supports the use of ORCID, and we encourage all coauthors to register for an ORCID iD and use it as well. Please encourage your coauthors to verify their ORCID iD within the submission system before final acceptance, as unverified ORCID iDs will not appear in the published article. *Only*  the individual author can complete the verification step; PLOS staff *cannot*  verify ORCID iDs on behalf of authors.

We look forward to receiving your revised manuscript.

Kind regards,

Alison Parker

Academic Editor

PLOS One

Journal Requirements:

If the reviewer comments include a recommendation to cite specific previously published works, please review and evaluate these publications to determine whether they are relevant and should be cited. There is no requirement to cite these works unless the editor has indicated otherwise.

**Additional Editor Comments:**

Thanks for your edits so far.   You've made good progress so far but reviewer 1 has highlighted some points that still need addressing.

[Note: HTML markup is below. Please do not edit.]

Reviewers' comments:

Reviewer's Responses to Questions

**Comments to the Author**

1. If the authors have adequately addressed your comments raised in a previous round of review and you feel that this manuscript is now acceptable for publication, you may indicate that here to bypass the “Comments to the Author” section, enter your conflict of interest statement in the “Confidential to Editor” section, and submit your "Accept" recommendation.

Reviewer #1: All comments have been addressed

**********

2. Is the manuscript technically sound, and do the data support the conclusions?

The manuscript must describe a technically sound piece of scientific research with data that supports the conclusions. Experiments must have been conducted rigorously, with appropriate controls, replication, and sample sizes. The conclusions must be drawn appropriately based on the data presented.

Reviewer #1: Partly

**********

3. Has the statistical analysis been performed appropriately and rigorously?

Reviewer #1: Yes

**********

4. Have the authors made all data underlying the findings in their manuscript fully available?

The PLOS Data policy requires authors to make all data underlying the findings described in their manuscript fully available without restriction, with rare exception (please refer to the Data Availability Statement in the manuscript PDF file). The data should be provided as part of the manuscript or its supporting information, or deposited to a public repository. For example, in addition to summary statistics, the data points behind means, medians and variance measures should be available. If there are restrictions on publicly sharing data—e.g. participant privacy or use of data from a third party—those must be specified.

Reviewer #1: Yes

**********

5. Is the manuscript presented in an intelligible fashion and written in standard English?

PLOS ONE does not copyedit accepted manuscripts, so the language in submitted articles must be clear, correct, and unambiguous. Any typographical or grammatical errors should be corrected at revision, so please note any specific errors here.

Reviewer #1: Yes

**********

6. Review Comments to the Author

Please use the space provided to explain your answers to the questions above. You may also include additional comments for the author, including concerns about dual publication, research ethics, or publication ethics. (Please upload your review as an attachment if it exceeds 20,000 characters)

Reviewer #1: Thank you for your revision. Here are additional areas for improvement.

Methodological Transparency: While you have added detail on sampling, recruitment, and translation, the reporting still feels uneven. For example, the justification for combining purposive and convenience sampling remains weak, and the explanation of data saturation is somewhat formulaic rather than demonstrating how saturation was assessed in practice.

Reflexivity: The reflexive practices described (journaling, team discussions) are mentioned but not sufficiently illustrated with examples of how they influenced analytic decisions. This risks reflexivity being treated as a checklist rather than an integrated practice.

Findings Presentation: Although more participant quotations have been added, some themes still read as researcher-driven interpretations rather than grounded accounts. For instance, the framing of “social surveillance” and “visibility” feels imposed rather than emerging directly from participant narratives.

Policy Implications: The discussion now avoids unsupported claims (e.g., climate resilience), but the recommendations remain somewhat generic. Stronger linkage between specific findings (e.g., inadequate WASH facilities, stigma in schools) and actionable interventions would make the policy relevance sharper.

**********

7. PLOS authors have the option to publish the peer review history of their article (what does this mean?). If published, this will include your full peer review and any attached files.

If you choose “no”, your identity will remain anonymous but your review may still be made public.

**Do you want your identity to be public for this peer review?**  For information about this choice, including consent withdrawal, please see our Privacy Policy.

Reviewer #1: No

**********

[NOTE: If reviewer comments were submitted as an attachment file, they will be attached to this email and accessible via the submission site. Please log into your account, locate the manuscript record, and check for the action link "View Attachments". If this link does not appear, there are no attachment files.]

To ensure your figures meet our technical requirements, please review our figure guidelines: https://journals.plos.org/plosone/s/figures

You may also use PLOS’s free figure tool, NAAS, to help you prepare publication quality figures: https://journals.plos.org/plosone/s/figures#loc-tools-for-figure-preparation.

NAAS will assess whether your figures meet our technical requirements by comparing each figure against our figure specifications.

# Author comment: Author response to Decision Letter 2

**DOI:** 10.1371/journal.pone.0354260.r004

2 Jul 2026

Detailed Response to Reviewers

Journal: PLOS One

Article type: Original Research Article

Manuscript title: Unspoken Cycles: A Qualitative Study Exploring the Lived Experiences of Women’s Menstruation in the Urban Informal Settlements of Bangladesh

Submission ID: PONE-D-26-09182R1

Comments from Reviewer-01

Thank you for your revision. Here are additional areas for improvement.

1. Methodological Transparency: While you have added detail on sampling, recruitment, and translation, the reporting still feels uneven. For example, the justification for combining purposive and convenience sampling remains weak, and the explanation of data saturation is somewhat formulaic rather than demonstrating how saturation was assessed in practice.

Author Response:

Thank you for this valuable suggestion. We have substantially strengthened the Sampling and Data Collection subsection. These revisions have been incorporated into the Methods section.

(Lines: 254-265, and 294-307)

First, we now provide a clearer justification for combining purposive and convenience sampling. Purposive sampling was employed to intentionally recruit women and adolescent girls who met the predefined eligibility criteria and possessed direct experience of menstruation in informal settlements. Convenience sampling complemented this strategy because participant recruitment occurred within a resource-constrained urban slum where participant availability, household responsibilities, and willingness to participate varied considerably. Combining both approaches enabled us to access information-rich participants while accommodating practical constraints encountered during fieldwork.

Second, we have expanded the description of data saturation. Rather than simply stating that saturation was achieved, we now explain how saturation was assessed throughout the analytic process. After the fifteenth interview, no substantially new codes or conceptual categories emerged from the data. Three additional interviews were conducted to confirm that existing themes remained consistent and that no new insights were identified. This iterative process provided confidence that thematic saturation had been achieved.

2. Reflexivity: The reflexive practices described (journaling, team discussions) are mentioned but not sufficiently illustrated with examples of how they influenced analytic decisions. This risks reflexivity being treated as a checklist rather than an integrated practice.

Author Response: We appreciate this insightful observation. To address this concern, we have expanded the reflexivity section by providing concrete examples of how reflexive practice informed our analytical decisions throughout the study. (Lines: 342-366)

Specifically, we explain that during the early stages of coding, researchers initially interpreted participants' descriptions of avoiding public spaces during menstruation primarily as consequences of inadequate sanitation infrastructure. However, through reflexive journaling and regular discussions among the research team, we recognized that these experiences also reflected internalized stigma, social expectations, and gendered surveillance within the community. Consequently, the coding framework was refined to distinguish structural constraints from socio-cultural mechanisms of exclusion. We also clarify that reflexive discussions helped challenge researchers' assumptions and ensured that interpretations remained grounded in participants' accounts rather than researchers' prior expectations.

3. Findings Presentation: Although more participant quotations have been added, some themes still read as researcher-driven interpretations rather than grounded accounts. For instance, the framing of “social surveillance” and “visibility” feels imposed rather than emerging directly from participant narratives.

Author Response: Thank you for this valuable observation. We carefully reviewed the Findings section to ensure that the analysis remains firmly grounded in participants' lived experiences.

(Please have a look at the Findings part now)

In response to this comment, we revised sections where the narrative relied too heavily on researcher interpretation or abstract conceptual language. We strengthened the connection between participants' accounts and the analytical interpretation by incorporating additional verbatim quotations, reducing interpretive statements that were insufficiently supported by the data, and rewriting several transitional and summary paragraphs to foreground participants' own words and experiences.

Where participants described feelings of embarrassment, fear of being judged, the need to conceal menstruation, restrictions on mobility, or difficulties accessing menstrual products, these accounts are now presented first, with analytical interpretation introduced only after sufficient empirical evidence has been established. We also revised several descriptive passages to use participant-centered language (e.g., "participants described...", "women reported...", and "their accounts suggest...") rather than researcher-driven assertions.

4. Policy Implications: The discussion now avoids unsupported claims (e.g., climate resilience), but the recommendations remain somewhat generic. Stronger linkage between specific findings (e.g., inadequate WASH facilities, stigma in schools) and actionable interventions would make the policy relevance sharper.

Author Response: Thank you for this valuable suggestion. We have substantially revised the Policy Implications section to establish a clearer and more explicit connection between our empirical findings and the recommended interventions. Rather than presenting broad recommendations, each policy implication is now directly informed by specific findings of the study. (Lines: 682-727)

For example, participants' experiences of inadequate access to private toilets, clean water, and menstrual waste disposal facilities are now directly linked to recommendations for strengthening gender-responsive WASH infrastructure in informal settlements and schools. Similarly, findings related to menstrual stigma, household silence, misinformation, and school absenteeism have been connected to specific interventions, including integrating comprehensive menstrual health education into school curricula, teacher sensitization, community awareness programs involving parents and men, and the establishment of menstrual-friendly school environments. Recommendations addressing the affordability of menstrual products are now explicitly based on participants' accounts of financial hardship and reliance on reusable cloth, leading to proposals for subsidized menstrual products, community-based distribution mechanisms, and support for locally produced reusable menstrual products. We also strengthened the recommendations by identifying relevant implementation stakeholders, including local government authorities, educational institutions, NGOs, community health workers, and development partners, making the proposed interventions more practical and contextually relevant for informal settlements in Bangladesh.

We sincerely thank you for your constructive comments, which have helped us strengthen the manuscript. We look forward to your kind consideration of our revised submission.

Sincerely,

Md. Al-Mamun

Research Associate

BRAC Institute of Governance and Development (BIGD), BRAC University

Dhaka-1212, Bangladesh

Cell Phone Number: +8801797450408

Email: md.mamun@bracu.ac.bd or mamunsiraji6576@gmail.com

ORCID iD: https://orcid.org/0000-0002-4133-757X

Attachment Submitted filename: Response to Reviewers-03-07-26.docx

# Peer review: Decision Letter 2

**DOI:** 10.1371/journal.pone.0354260.r005

**Authors:** Alison Parker

6 Jul 2026

Unspoken Cycles: A Qualitative Study Exploring the Lived Experiences of Women’s Menstruation in the Urban Informal Settlements of Bangladesh

PONE-D-26-09182R2

Dear Dr. Al-Mamun,

We’re pleased to inform you that your manuscript has been judged scientifically suitable for publication and will be formally accepted for publication once it meets all outstanding technical requirements.

Within one week, you’ll receive an e-mail detailing the required amendments. When these have been addressed, you’ll receive a formal acceptance letter and your manuscript will be scheduled for publication.

An invoice will be generated when your article is formally accepted. Please note, if your institution has a publishing partnership with PLOS and your article meets the relevant criteria, all or part of your publication costs will be covered. Please make sure your user information is up-to-date by logging into Editorial Manager at Editorial Manager® and clicking the ‘Update My Information' link at the top of the page. For questions related to billing, please contact billing support.

If your institution or institutions have a press office, please notify them about your upcoming paper to help maximize its impact. If they’ll be preparing press materials, please inform our press team as soon as possible -- no later than 48 hours after receiving the formal acceptance. Your manuscript will remain under strict press embargo until 2 pm Eastern Time on the date of publication. For more information, please contact onepress@plos.org.

Kind regards,

Alison Parker

Academic Editor

PLOS One

Additional Editor Comments (optional):

Reviewers' comments:

Reviewer's Responses to Questions

**Comments to the Author**

1. If the authors have adequately addressed your comments raised in a previous round of review and you feel that this manuscript is now acceptable for publication, you may indicate that here to bypass the “Comments to the Author” section, enter your conflict of interest statement in the “Confidential to Editor” section, and submit your "Accept" recommendation.

Reviewer #1: All comments have been addressed

**********

2. Is the manuscript technically sound, and do the data support the conclusions?

The manuscript must describe a technically sound piece of scientific research with data that supports the conclusions. Experiments must have been conducted rigorously, with appropriate controls, replication, and sample sizes. The conclusions must be drawn appropriately based on the data presented.

Reviewer #1: Yes

**********

3. Has the statistical analysis been performed appropriately and rigorously?

Reviewer #1: Yes

**********

4. Have the authors made all data underlying the findings in their manuscript fully available?

The PLOS Data policy requires authors to make all data underlying the findings described in their manuscript fully available without restriction, with rare exception (please refer to the Data Availability Statement in the manuscript PDF file). The data should be provided as part of the manuscript or its supporting information, or deposited to a public repository. For example, in addition to summary statistics, the data points behind means, medians and variance measures should be available. If there are restrictions on publicly sharing data—e.g. participant privacy or use of data from a third party—those must be specified.

Reviewer #1: Yes

**********

5. Is the manuscript presented in an intelligible fashion and written in standard English?

PLOS ONE does not copyedit accepted manuscripts, so the language in submitted articles must be clear, correct, and unambiguous. Any typographical or grammatical errors should be corrected at revision, so please note any specific errors here.

Reviewer #1: Yes

**********

6. Review Comments to the Author

Please use the space provided to explain your answers to the questions above. You may also include additional comments for the author, including concerns about dual publication, research ethics, or publication ethics. (Please upload your review as an attachment if it exceeds 20,000 characters)

Reviewer #1: The revisions you made in response to reviewer comments have strengthened the methodological transparency, particularly in clarifying the rationale for combining purposive and convenience sampling and in detailing how saturation was assessed. The reflexivity section is now more robust, with concrete examples that demonstrate how team discussions and journaling shaped analytic decisions. Additionally, the Findings section is better grounded in participant narratives, with verbatim quotations foregrounded before interpretation. The policy implications are clearer and more directly linked to empirical findings, which enhances the practical relevance of your work. Overall, the manuscript is significantly improved, though continued attention to maintaining participant-centered language throughout will ensure the analysis remains firmly rooted in lived experiences.

**********

7. PLOS authors have the option to publish the peer review history of their article (what does this mean?). If published, this will include your full peer review and any attached files.

If you choose “no”, your identity will remain anonymous but your review may still be made public.

**Do you want your identity to be public for this peer review?**  For information about this choice, including consent withdrawal, please see our Privacy Policy.

Reviewer #1: No

**********

# Editor report: Acceptance letter

**DOI:** 10.1371/journal.pone.0354260.r006

**Authors:** Alison Parker

PONE-D-26-09182R2

PLOS One

Dear Dr. Al-Mamun,

I'm pleased to inform you that your manuscript has been deemed suitable for publication in PLOS One. Congratulations! Your manuscript is now being handed over to our production team.

At this stage, our production department will prepare your paper for publication. This includes ensuring the following:

* All references, tables, and figures are properly cited

* All relevant supporting information is included in the manuscript submission,

* There are no issues that prevent the paper from being properly typeset

You will receive further instructions from the production team, including instructions on how to review your proof when it is ready. Please keep in mind that we are working through a large volume of accepted articles, so please give us a few days to review your paper and let you know the next and final steps.

Lastly, if your institution or institutions have a press office, please let them know about your upcoming paper now to help maximize its impact. If they'll be preparing press materials, please inform our press team within the next 48 hours. Your manuscript will remain under strict press embargo until 2 pm Eastern Time on the date of publication. For more information, please contact onepress@plos.org.

You will receive an invoice from PLOS for your publication fee after your manuscript has reached the completed accept phase. If you receive an email requesting payment before acceptance or for any other service, this may be a phishing scheme. Learn how to identify phishing emails and protect your accounts at https://explore.plos.org/phishing.

If we can help with anything else, please email us at customercare@plos.org.

Thank you for submitting your work to PLOS ONE and supporting open access.

Kind regards,

PLOS ONE Editorial Office Staff

on behalf of

Dr. Alison Parker

Academic Editor

PLOS One
